# Plasma Metabolomic Alterations Induced by COVID-19 Vaccination Reveal Putative Biomarkers Reflecting the Immune Response

**DOI:** 10.3390/cells11071241

**Published:** 2022-04-06

**Authors:** Ioanna Dagla, Aikaterini Iliou, Dimitra Benaki, Evagelos Gikas, Emmanuel Mikros, Tina Bagratuni, Efstathios Kastritis, Meletios A. Dimopoulos, Evangelos Terpos, Anthony Tsarbopoulos

**Affiliations:** 1 The Goulandris Natural History Museum, Bioanalytical Laboratory, GAIA Research Center, 145 62 Kifissia, Greece; idagla@pharm.uoa.gr; 2 Division of Pharmaceutical Chemistry, Faculty of Pharmacy, School of Health Sciences, National and Kapodistrian University of Athens, Panepistiomiopolis, Zografou, 157 71 Athens, Greece; katerinail@pharm.uoa.gr (A.I.); dbenaki@pharm.uoa.gr (D.B.); 3 Laboratory of Analytical Chemistry, Faculty of Chemistry, National and Kapodistrian University of Athens, Panepistimioupolis, Zografou, 157 71 Athens, Greece; vgikas@chem.uoa.gr; 4 Department of Clinical Therapeutics, School of Medicine, National and Kapodistrian University of Athens, Panepistiomiopolis, Zografou, 115 28 Athens, Greece; tbagratuni@med.uoa.gr (T.B.); ekastritis@med.uoa.gr (E.K.); mdimop@med.uoa.gr (M.A.D.); eterpos@med.uoa.gr (E.T.); 5 Department of Pharmacology, Medical School, National and Kapodistrian University of Athens, Panepistiomiopolis, Zografou, 115 27 Athens, Greece

**Keywords:** COVID-19, metabolomics, neutralizing antibodies (NAbs), NMR, LC-MS, ceramides

## Abstract

Vaccination is currently the most effective strategy for the mitigation of the COVID-19 pandemic. mRNA vaccines trigger the immune system to produce neutralizing antibodies (NAbs) against SARS-CoV-2 spike proteins. However, the underlying molecular processes affecting immune response after vaccination remain poorly understood, while there is significant heterogeneity in the immune response among individuals. Metabolomics have often been used to provide a deeper understanding of immune cell responses, but in the context of COVID-19 vaccination such data are scarce. Mass spectrometry (LC-MS) and nuclear magnetic resonance (NMR)-based metabolomics were used to provide insights based on the baseline metabolic profile and metabolic alterations induced after mRNA vaccination in paired blood plasma samples collected and analysed before the first and second vaccination and at 3 months post first dose. Based on the level of NAbs just before the second dose, two groups, “low” and “high” responders, were defined. Distinct plasma metabolic profiles were observed in relation to the level of immune response, highlighting the role of amino acid metabolism and the lipid profile as predictive markers of response to vaccination. Furthermore, levels of plasma ceramides along with certain amino acids could emerge as predictive biomarkers of response and severity of inflammation.

## 1. Introduction

The spread of the severe acute respiratory syndrome coronavirus 2 (SARS-CoV-2) has led to the worldwide COVID-19 pandemic. It has infected more than 409 million individuals worldwide and it is responsible for more than 5.8 million deaths to date (https://www.worldometers.info/coronavirus/, accessed on 12 February 2022). These numbers are increasing rapidly every day and have caused an unprecedented global health crisis. Vaccination is probably the most effective strategy to mitigate the effects of the pandemic. Several vaccines have been approved by the Food and Drug Administration (FDA) to prevent life-threatening COVID-19 (coronavirus disease 2019) (https://www.cdc.gov/coronavirus/2019-ncov/vaccines/different-vaccines.html, accessed on 12 February 2022). Messenger RNA (mRNA) vaccine technology has only recently become available for public use, even though scientists have been studying mRNA vaccines for decades [1]. Currently the two approved COVID-19 mRNA vaccines code the production of the “spike” protein that the SARS-CoV-2 virus uses to enter cells. This protein triggers the immune system to produce antibodies and activate other immune cells [2]. According to the Center for Disease Control and Prevention (CDC), two 21-day interval doses of mRNA vaccines are required in order to achieve immune protection against SARS-CoV-2. Due to the fact that the vaccine effectiveness declines, a third booster dose is also required in order to maintain the immunity.

A study of the kinetics of anti-SARS-CoV-2 antibody responses three months after complete vaccination with BTN162b2, which is an mRNA vaccine developed by Pfizer-BioNTech, showed that neutralizing antibodies (NAbs) increase until the 36th day from the 1st vaccine shot, and slightly decrease thereafter. A similar pattern is observed for anti-SARS-CoV-2 Spike-receptor binding domain (anti-S-RBD) antibodies [3]. It appears that the highest NAbs production reaches a maximum level 14 days after the second vaccination, with a slow decline in NAbs levels thereafter. In the same study, it was observed that the level of NAbs titers vary significantly from approximately 1 to 100% (median 53.8%) 21 days after the first shot and before the second vaccine dose. Among the factors that were shown to affect the immunization efficacy against SARS-CoV-2 with the BTN162b2 vaccine were age and gender; aged people were more likely to exhibit a reduced response, while female subjects had higher response [4]. However, the underlying molecular processes that affect immune response after mRNA vaccines against SARS-CoV-2 remain poorly understood. The innate and adaptive immune response to vaccines, such as activation of immune cells and antibody formation, often require a metabolic reprogramming and can affect central metabolic processes [5]. For this scope, use of metabolomics was deemed indispensable. Metabolomics—the systematic analysis of small molecules (50 to 1500 Da) e.g., carbohydrates, amino acids and lipids produced by regulatory mechanisms and during cellular processes or from exogenous sources (e.g., diet and drugs)—is a powerful tool for biomarker discovery, prediction of therapy response and investigation of the biochemical mechanisms concerning several human diseases, aiming to improve their diagnosis and prevention, and to design better therapeutic strategies [6,7,8,9]. The two most frequently used analytical techniques in metabolomics are mass spectrometry (MS) and nuclear magnetic resonance (NMR) spectroscopy [10]. These techniques are complementary and each one brings both advantages and limitations [11]. The combination of information from these complementary analytical platforms facilitates the detection and identification of metabolites that could not be discovered by a single technique thereof.

Since the outbreak of the COVID-19 pandemic, a limited number of metabolomics studies have been performed in COVID-19 patients, indicating a distinct metabolic profile associated with COVID-19 infection and/or disease severity [12]. Thus, an LC-MS based metabolomics study showed perturbations in the amino acid and the kynurenine pathway in COVID-19 patients compared to healthy controls, while these alterations also correlated with concentrations of pro-inflammatory cytokines and chemokines [13]. However, metabolomic studies associated with the immunity induced by vaccination against SARS-CoV-2 are still lacking. To the best of our knowledge, there is only one study in this field that highlighted the metabolic networks associated with the immune responses induced by the CoronaVac, an inactivated vaccine against COVID-19, showing that metabolites involved in the tricarboxylic acid (TCA) cycle and amino acid metabolism were associated with immunity [14]. Nevertheless, there is still no evidence for the metabolic profiles associated with the novel mRNA vaccines against SARS-CoV-2.

In this study, a combination of NMR and LC-MS metabolomics were employed in order to provide insights concerning alterations in the metabolic profiles of human subjects in association with the antibody levels induced by BTN162b2 vaccination and predict the vaccination efficacy. Metabolic characterization was performed using plasma samples of individuals, divided into two groups: the “high responders” including individuals with strong immune response after the first dose of vaccination with BTN162b2, as measured by the levels of NAbs (*n* = 29) and the “low responders” (*n* = 29) showing limited response to the first vaccine dose with BTN162b2. A combination of both NMR and LC-MS strategies was adopted in order to gain the maximum amount of information. Our goal was to define metabolic fingerprints and discover putative biomarkers of immune response to BTN162b2 that will allow us to predict a-priori reduced response to the vaccination from Day 1. The metabolomic alterations that were identified can highlight predictive biomarkers for the prediction of response and severity of inflammation, enabling the design of more effective strategies towards increasing our protection against SARS-CoV-2.

## 2. Materials and Methods

### 2.1. Plasma Samples

Plasma samples (stored at −80 °C) of 58 healthy individuals after vaccination with the BNT162b2 COVID-19 vaccine on days 1 (before the first vaccine shot), day 22 (before the second vaccine shot) and at 90 days (3 months after the first vaccine shot) were analysed. Samples were selected based on the level of NAbs against SARS-CoV-2 at Day 22 and were discriminated into A. “low responders” (NAbs < 40%, *n* = 29) and B. “high responders” (Nabs > 70%, *n* = 29). Participants aged from 25 to 68 years old; 40 of them were women (*n*_LOW_ = 17, *n*_HIGH_ = 23) and 18 were men (*n*_LOW_ = 12, *n*_HIGH_ = 6). 

### 2.2. Chemicals and Reagents

The chemicals that were used for NMR and UPLC-MS analysis are described in Supplementary Materials S2 (Section S1.1).

### 2.3. NAbs Measurements

SARS-CoV-2 NAbs were measured using the FDA-approved cPass^TM^ SARS-CoV2 NAbs Detection Kit (GenScript, Piscataway, NJ, USA). Values were provided as percentage of SARS-CoV-2 binding inhibition. According to the FDA, a high NAbs titer for this specific method is considered any value above or equal to 68%.

### 2.4. Plasma Sample Preparation

The plasma sample preparation is described in Supplementary Materials S2 (Sections S1.2.1 and S1.2.2 for NMR and UPLC-MS analyses, respectively).

### 2.5. Data Acquisition

All NMR experiments were performed using the NMR Bruker AVANCE III 600 MHz Spectrometer (Bruker BioSpin GmbH, Karlsruhe, Germany). Details are presented in Supplementary S2 (Section S1.3.1).

For the MS analysis, a Waters ESI-QTOF Premier mass spectrometer (Waters Corp., Milford, CT, USA) coupled to an Acquity UPLCTM system (Waters Corp., Milford, MA, USA) was used. Details are presented in Supplementary Materials S2 (Section S1.3.2).

### 2.6. Data Processing

The NMR and MS data processing is described in detail in Supplementary Materials S2 (Sections S1.4.1 and S1.4.2, respectively). Detailed description of the “SMolESY” (Small Molecule Enhancement SpectroscopY) algorithm is provided in [15].

### 2.7. Study Approval

All participants were enrolled in a large prospective study (NCT04743388) evaluating the kinetics of anti-SARS-CoV-2 antibodies after COVID-19 vaccination in healthy subjects. The study was approved by the Institutional Ethics Committee of General Hospital Alexandra, Athens, Greece (Institutional Review Board Protocol ID: 900/24-12-2020) in accordance with the Declaration of Helsinki and the International Conference on Harmonization for Good Clinical Practice. All participating individuals provided written informed consent prior to enrolment in the study.

## 3. Results

### 3.1. Cohort Characteristics

The demographic characteristics of the 58 healthy individuals and the comparison of the characteristics of the two groups (“high” and “low” responders) included in this study are shown in Table 1. At Day 1, the NAbs were low for all participants as expected, while they reach a high level for all participants in the third month. Relatively high levels of NAbs (>30%) were observed in two participants at Day 1, putatively due to previous infection. However, the “high responders” group exhibits higher levels of NAbs (mean, median, 1st and 2nd quantiles) at all time points. Detailed NAbs measurements of all individuals are provided in Table S1.

### 3.2. NMR Results

#### 3.2.1. Metabolite Identification

In total, 41 known metabolites were identified in the plasma NMR spectra including several amino acids, organic acids, alcohols and sugars involved in core biochemical processes. Detailed chemical shifts of all annotated metabolites and respective multiplicity of peaks are provided in Table S2, while spectral annotation is also shown in Figure S1.

#### 3.2.2. Correlation of NMR Metabolic Features with Immune Response

**Figure 1:**
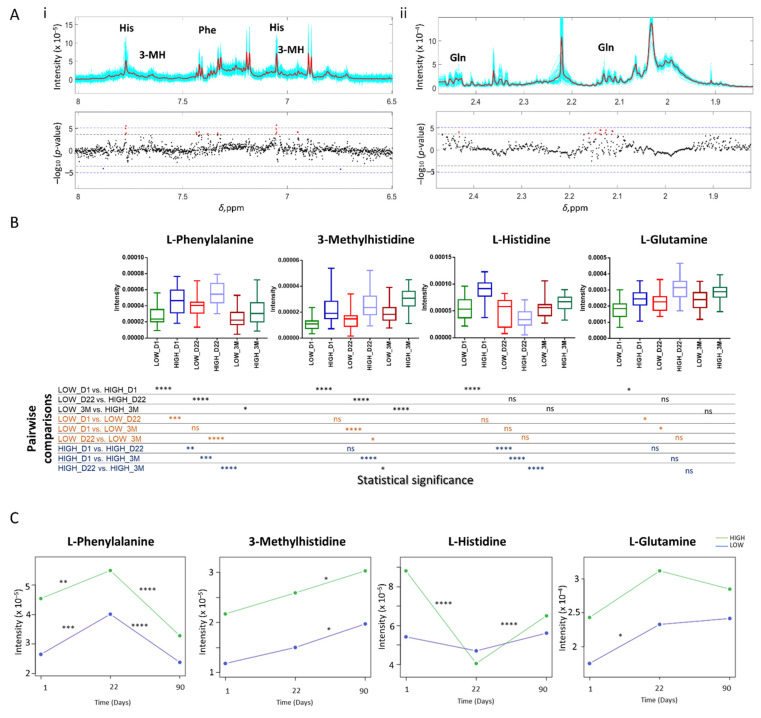
Correlation of NMR metabolic features with immune response. (**A**) Manhattan-type plot showing the analysis of the 6555 CPMG NMR features with response at day 22. The signed −log_10_(*p*-value) is derived from Spearman correlation analysis with adjustment for age and sex. The dotted lines represent the threshold after multiple Bonferroni testing correction (blue line, *p*-value < 1.8 × 10^−5^) or FDR (black, *p*-value < 2.3 × 10^−4^), and the red dots represent the data points that remain significant after FDR. The horizontal axis is the NMR chemical shift (in ppm). In the upper panel, the 58 spectra (cyan) and their means (red) are depicted. Associations with (**i**) l-histidine (His), l-phenylalanine (Phe) and 3-methylhistidine (3-MH) and (**ii**) l-Glutamine (Gln) are shown. (**B**) Boxplots of the significant metabolites at all the 3 time points. In black, comparison of low vs. high responders within each time point. In orange, comparison between the three time points in the low responders group. In blue, comparison between the three time points in the high responders group. (**C**) Longitudinal change of the significant metabolites among the three time points. Asterisks indicate statistical significance using ANOVA: **** *p* < 0.0001, *** 0.0001 < *p* < 0.001, ** 0.001 < *p* < 0.01, * 0.01 < *p* < 0.05, ns: no significance.

Spearman correlation between the NMR metabolic features measured on the first day of vaccination with the levels of NAbs after 22 days revealed an association of baseline metabolite levels with the vaccine-induced immune response. The data were adjusted for age and gender and highlighted a panel of metabolites, including mainly amino acids, that were related to vaccination efficacy, independently of the two major confounders (age and gender). In more details, out of the 6555 examined 1D CPMG NMR spectral bins reflecting the levels of low molecular weight metabolites, 33 were significant at FDR 95% (*p*-value < 2.29 × 10^−4^) (Figure 1A).

Among these, 27 were assigned to known metabolites including l-histidine, l-phenylalanine, l-glutamine and 3-methylhistidine (3-ΜH) (Table 2).

Metabolic features assigned to l-histidine (spectral bins *δ* 7.772 and *δ* 7.052) remained significant even after applying stricter criteria for multiple hypothesis testing using the Bonferroni correction (*p*-value < 7.63 × 10^−6^) (Figure 1A(i,ii), blue dotted line). Detailed results of all the associated spectral bins at FDR 95%, along with assignment, correlation coefficients and *p*-values are provided in Table S3. Other amino acids also showed a strong association with response but did not reach the level of statistical significance after correction for multiple hypothesis testing; a positive association was observed for glycine (*p*-value = 0.0012) and l-lysine (*p*-value = 8.9 × 10^−4^), while a negative association was observed for l-valine (*p*-value = 0.0013). Figure S2 shows the strength and direction of effect of the annotated metabolites on the examined outcome (i.e., the response to vaccination). Glutamic acid showed an inverse association with glutamine levels (Figure S2, l-Glutamic acid and l-Glutamine respectively).

We further examined the alterations in l-histidine, l-phenylalanine, l-glutamine and 3-MH levels in the two examined groups at all 3 time points using the spectral bin exhibiting the lowest *p*-value in the correlation analysis (Figure 1B). All four metabolites were positively associated with the response to vaccination. l-phenylalanine and 3-MH levels were significantly increased in high responders compared to low responders at all time points, although for l-phenylalanine the difference was slightly decreased at 3 months. Moreover, although l-phenylalanine levels increased in both groups at Day 22, they declined at 3 months. l-Histidine and l-glutamine were significantly increased in the high responders compared to the low responders only at Day 1. l-histidine levels were also changing across the 3 time points in the high responders, while no significant alterations were observed in the low responders (Figure 1C). On the other hand, l-glutamine levels were significantly increased in the low responders’ group at Day 22 and 3 months compared to Day 1.

#### 3.2.3. NMR Targeted Metabolite Quantification

**Figure 2:**
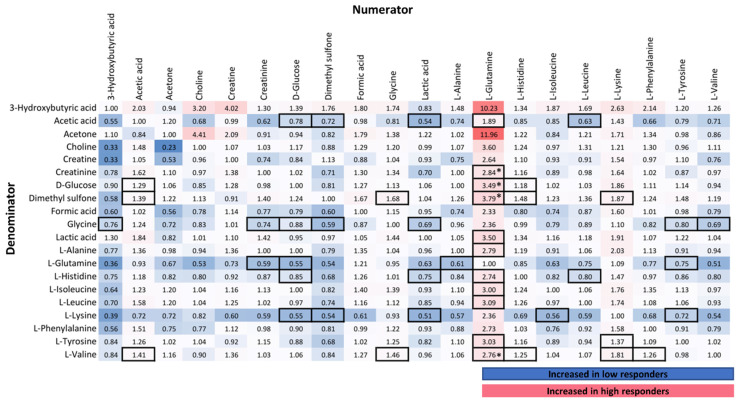
Fold-change of high vs. low responders of metabolic ratios in Day 1. Columns show the numerator and rows the denominator of each ratio. For example, the l-Glutamine/d-Glucose ratio exhibits a 3.49-fold increase in the high responders compared to the low responders. The changes in the metabolic ratios found statistically significant using *t*-tests (*p*-value < 0.05) are in boxes. Asterisks (*) indicate statistical significance after FDR correction (*p*-value < 4.62 × 10^−4^). Color coding is based on conditional formatting of fold-change values.

We further quantified 22 metabolites in the NOESY spectrum using the “SMolESY-select” platform, which enhances the resolution of small molecules. Citric acid (used as an anticoagulant) and ethanol (only observed in a few samples) were excluded from analysis. To normalize the effects of analytical variation in the data, we calculated the metabolic ratios of these metabolites (20 × 20 = 400 metabolic traits). In Figure 2, the fold changes between high vs. low responders’ group at Day 1 for each of the 400 traits are provided in a table format, where columns depict the numerator and rows the denominator of each ratio. Metabolites that emerged as significant included l-glutamine, glycine, acetic acid, l-histidine, and l-lysine. Significant ratios with amino acids in the numerator were increased in the high responders’ group (FC > 1), in accordance with the findings from the untargeted correlation of NMR spectral bins with NAb levels. Four metabolic ratios of glutamine remained significant using FDR correction (*p*-value < 4.62 × 10^−4^), highlighting again the role of glutamine in immune responses. These ratios included increased glutamine/valine (*p*-value = 5.29 × 10^−5^), glutamine/creatinine (*p*-value = 9.52 × 10^−5^), glutamine/glucose (*p*-value = 1.09 × 10^−4^) and glutamine/dimethyl sulfone (*p*-value = 3.48 × 10^−4^) ratios in the high responders, indicating elevated glutamine levels in this group. Other metabolites that were significant as ratios with the aforementioned metabolites included d-glucose, l-lactic acid, dimethyl sulfone and l-tyrosine. The l-phenylalanine to l-valine ratio was also increased in the high responders. The respective metabolic alterations in Day 22 and 3 months are shown in Figure S3. At 3 months, metabolic ratios of l-histidine, l-glutamine and l-lysine in the numerator were significantly increased in the high responders’ group. At Day 22, l-histidine emerged again as an important metabolite (8 out of the 19 ratios of l-histidine were significant), while the strongest increase was observed for acetone.

#### 3.2.4. Multivariate Analysis

**Figure 3:**
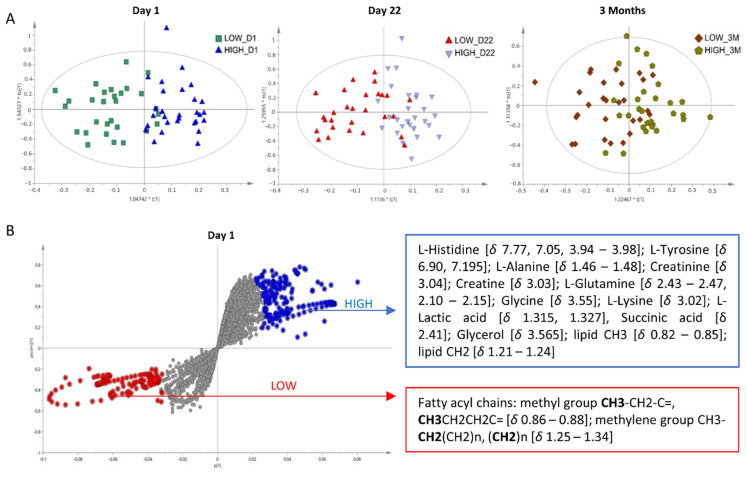
NMR Multivariate Analysis. (**A**) Scores plots obtained from the OPLS-DA analysis of NMR CPMG from plasma samples of high vs. low responders at Day 1, Day 22 and 3 Months. The two groups are discriminated in all time points, with the discrimination being clearer at Day 1 and Day 22. (**B**) S-plot obtained from the OPLS-DA of NMR CPMG from plasma samples of high vs. low responders after first dose at Day 1. Spectral variables on the top-right corner (blue) are considered significantly increased in the high responders, while those on the lower-left corner (red) are increased in the low responders. Metabolites with *p* and *p(corr)*~0 (colored in grey) do not significantly impact the separation. Significant spectral variables and their assigned metabolites are shown.

To take advantage of the global nature of metabolomics, we further exploited multivariate approaches to investigate distinct metabolic fingerprints in relation to response at each of the three examined time points. The unsupervised PCA analysis provided in Figure S4 showed a trend for discrimination between the two groups at Day 1 and to a lesser extent at Day 22, while this trend was not observed after three months. PCA offered an unbiased way to detect clusters of samples that share common variation. However, due to large inter-group variation observed in human studies (introduced by diverse lifestyle, dietary habits, smoking status, health and medication etc.), supervised methods were needed for optimized separations to be obtained. OPLS-DA analysis, which also eliminates the variation in the metabolomics data unrelated to the examined outcome, showed clear discrimination of low and high responders at all the examined time points, as shown in the cross-validated score plots (Figure 3A). The quality parameters and prediction power at the three time points were the following: Day 1: R^2^Y (cum) = 0.663 and Q^2^ (cum) = 0.384], Day 22: [R^2^Y (cum) = 0.569 and Q^2^ (cum) = 0.262]; 3 months: R^2^Y (cum) = [0.368 and Q^2^ (cum) = −0.104]. Interestingly, the obtained separation at Day 1 showed stronger statistical significance compared to the other time points, indicating that the response to the vaccine after the second dose could have already been predicted from the plasma metabolic fingerprint of the individual at Day 1. Poor quality parameters were obtained in the 3 Months model. Permutation testing (*n* = 100 permutations) provided in Figure S5 (Permutations) also proved the validity of the Day 1 and Day 22 models. On the other hand, permutation testing on the 3 Months model indicates low quality parameters of the model, highlighting that the observed metabolic differences were diminished after the second dose.

Detailed examination of the S-plot derived from the Day 1 OPLS-DA model was further conducted to highlight the metabolites with the highest discriminating power at Day 1 (Figure 3B). Spectral variables on the upper right corner (blue) were considered significantly increased in the “High responders” group and on the lower left corner (red) in the “Low responders” group. Assignment of these spectral features showed increased levels of lipid chain signals in the “Low responders” group. The high responders exhibited increased levels of several amino acids, including l-histidine, l-tyrosine, l-alanine, and l-glutamine; l-glycine and l-lysine, organic acids including succinic acid and l-lactic acid, creatinine, as well as increased intensities of certain fatty acyl lipid chains. Detailed information about the spectral variables contributing the most to separation and respective assignments are provided in Table S4. Information on lipid annotation is provided in Table S5. 

In order to examine whether the differences in the lipid profile obtained by the multivariate analysis were gender-dependent, we further conducted the OPLS-DA stratified by gender. Discrimination of female low- and high-responders was observed in the OPLS-DA scores plot (Figure S6A). The fatty acyl lipid chains and amino acids exhibited again the highest impact in the discrimination, based on the edges of the S-plot (Figure S6B). On the other hand, d-glucose emerged as strongly significant in this analysis, highlighting a gender-dependent impact for this metabolite. The model was validated through permutation testing (Figure S6C). Similar results were obtained in the male cohort (Figure S7). However, quality parameters of the model were poorer, putatively due to smaller sample size.

**Figure 4:**
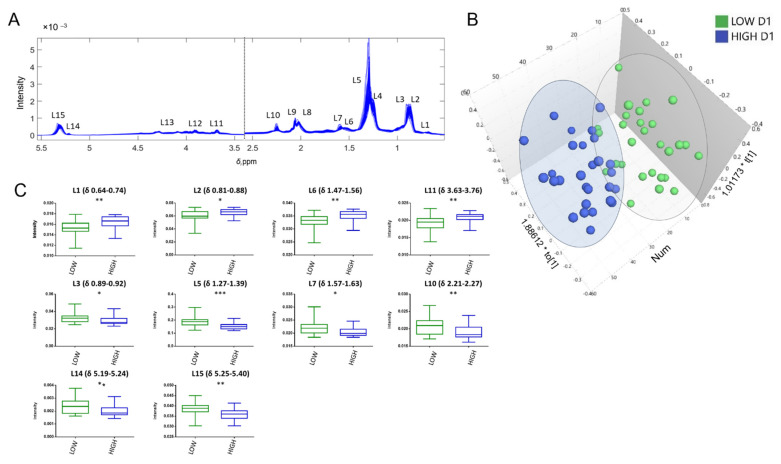
Analysis of longitudinal encode-decode (LED) spectra. (**A**) LED NMR spectra (*n* = 58) with assignment. (**B**) Scores plots obtained from the OPLS-DA of high vs. low responders at Day 1 after first dose. The two groups are discriminated into (●) Low responders at Day 1 and (●) High responders at Day 1. (**C**) Boxplots of the statistically significant lipids. The *p*-value of *t*-test is indicated on the top. Lipids numbering is according to Table S5. Asterisks indicate statistical significance: *** 0.0001 < *p* < 0.001, ** 0.001 < *p* < 0.01, * 0.01 < *p* < 0.05.

Due to the fact that several of the discriminative spectral variables in the OPLS-DA analysis of CPMG spectra were attributed to macromolecules, we further analysed our data with 1H NMR spectra recorded with a LED pulse sequence. The LED experiment reduces the intensities of the small molecules, resulting in a spectrum consisting majorly of the broad signals of the large molecules. Therefore, it is considered more appropriate for the investigation of lipoproteins and lipid changes. The results from the analysis of LED spectra are shown in Figure 4. Spectral annotation of lipid chemical moieties is provided in Figure 4A, as well as in Table S5. Low and high responders exhibited differences corresponding to lipoproteins and lipid moiety signals at Day 1, as shown in the scores plot of the OPLS-DA analysis (Figure 4B). Permutation testing using 100 random changes proved the validity of the model (Figure S8). T-testing of these spectral features revealed statistical significance for 11 out of the 16 examined macromolecule regions (Figure 4C). Information regarding signal assignment to each lipid class is provided in Figure S9 and is based on statistical spectroscopy and previous literature [16].

The glyceryl group CHOCOR (L14) from lipoprotein glycerophospholipids and triacylglycerols was increased in the low responders. Low responders also exhibited increased signals from unsaturated fatty acids including the methine group CH=CH (L15) and methylene group next to double bond -CH_2_-C=O (L10). These signals correlated with the =CHCH_2_CH= signal characteristic for the polyunsaturated fatty acids (PUFAs) (Figure S9B). On the other hand, signals from cholesterol (L1 and L11) (free or from cholesterol esters) were increased in the high responders. The signal of the choline head group from sphingomyelins, lysophosphocholines and phosphocholines was elevated in the high responder’s group but was excluded from analysis due to overlapping with EDTA region (Figure S9A). The peaks of the methyl groups (L2 vs. L3), the methylene groups (L4 vs. L5) and methylene groups next to double bonds (L6 vs. L7) in the different fatty acyl chains and lipids could not be resolved. Statistical spectroscopy and direction of effect between low and high responders showed that peaks L2, L4 and L6 correlate with cholesterol levels (L1) and are increased in the high responders (resembling the HDL spectrum), while peaks L3, L5 and L7, respectively, correlate with glycerophospholipids and triacylglycerols (L14) and are increased in the low responders. (Figure S9). Overall, these results indicate alterations in the lipid profile of high vs. low responders. However, the NMR peaks from different chains cannot be resolved and assigned to a distinct lipid or lipoprotein; thus, it is difficult to conclude to a single lipid biomarker.

### 3.3. LC–MS Results

#### 3.3.1. Multivariate Analysis

**Figure 5:**
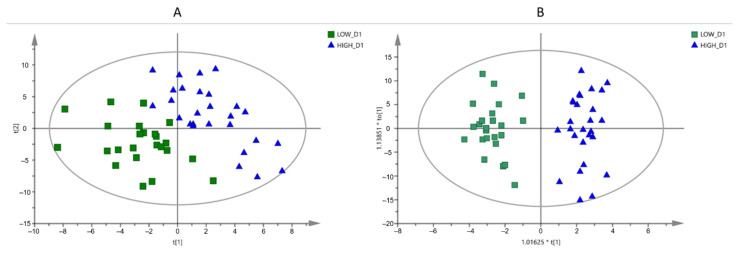
LC–MS Multivariate Analysis. (**A**) Scores plots obtained from the partial least squares-discriminant analysis of ESI (−) MS analysis from plasma samples of high vs. low responders after first dose at Day 1 and (**B**) Scores plots obtained from the orthogonal partial least squares-discriminant analysis of ESI (−) MS analysis from plasma samples of high vs. low responders after 1st dose at Day 1. Clear clustering is observed between low and high responders: (■) Low responders at Day 1; (▲) High responders at Day 1.

PCA frequently fails to produce any meaningful or appropriate clustering, as it also occurred in the current MS data. PCA-based approaches reveal separation between group sets only when the group variability is sufficiently greater than the variability within the group. In the current case, using the PLS-DA approach, a clear separation between the “low” and “high” responders’ groups was observed. The R^2^X, R^2^Y and Q^2^ values were 0.242, 0.863 and 0.238, respectively, employing 3 components in ESI (+), and 0.253, 0.853 and 0.247, respectively, employing 3 components in ESI (−). Permutation testing allowing 100 random permutations indicated that the PLS-DA models were reliable. The supervised OPLS-DA model was also employed to obtain even better discrimination between the two groups, as it separates predictive from orthogonal (non-predictive) variation. The two groups were clearly separated in both ion modes. The R^2^X, R^2^Y and Q^2^ values were 0.289, 0.957 and 0.317, respectively, in ESI (+), and 0.291, 0.92 and 0.224, respectively, in ESI (−). The PLS-DA and OPLS-DA models for ESI (−) are presented in Figure 5. Taking into consideration the variable importance in projection (VIP) scores and the S-plot, 15 features were selected as the most influential variables. Further efforts for the identification of features that contribute to the differentiation of the data at Day 1 between “low” and “high” responders were undertaken.

#### 3.3.2. Annotation of the Selected Variables

An example of the feature annotation workflow that was adopted in the current metabolomic study is presented below. The feature with *m*/*z* 634.6120 in ESI (−) and t_R_ = 18.99 min (ID: 634.6120_18.99) was detected in both MZmine and MS-Dial software. Compound annotation was performed, initially, by MS-Finder, which supports MS/MS spectra mining and provides formula prediction based on the isotope ratio and structure elucidation exploiting public spectral databases such as MassBank, LipidBlast, and GNPS. For the feature 634.6120_18.99, the formula C_41_H_81_NO_3_ had the higher score (score = 3.96/5.00, with Error = −0.33 mDa) and annotated as the ceramide (Cer) d18:1/23:0 according to the spectra matching (experimental vs. reference spectrum, score = 7.40/10). The *m*/*z* was searched also against HMDB, LIPID MAPS and MassBank for the verification of the results. Eventually, six compounds were annotated and presented in Table 3. The ROC curves of the annotated features are presented in Figure S10.

#### 3.3.3. Ceramides

**Figure 6:**
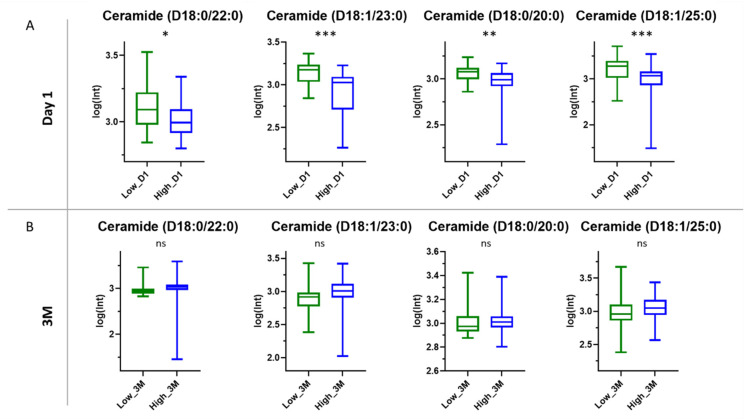
Box and whiskers plots of the detected ceramides for the low vs. high responders. (**A**) Day 1: The differences were statistically significant; *p*-values < 0.05. (**B**) three months after the first dose (3M): The differences were not statistically significant; *p*-values > 0.05. Asterisks indicate statistical significance: *** 0.0001 < *p* < 0.001, ** 0.001 < *p* < 0.01, * 0.01< *p* < 0.05; ns: not significant.

Among the six annotated compounds, four belonged to the ceramide class. It was observed that the levels of the detected ceramides were lower in the high responders at Day 1 than in the low responders (Figure 6A). 

The differences between the two groups were statistically significant according to the results of the *t*-test; *p*-values were equal to 0.020, 0.003, 0.005 and 0.001 for Cer (D18:0/22:0), Cer (D18:1/23:0), Cer (D18:0/20:0) and Cer (D18:1/25:0), respectively. Examining the levels of ceramides between the genders, no statistically significant difference was observed (*p* > 0.05) at Day 1 for all ceramides (Figure S11). In order to investigate the levels of those ceramides after the vaccination, further data analysis was undertaken. The levels of the four ceramides were identified in the LC-MS data after 3 months. Our study revealed that 22 days after the first dose, the levels of ceramides were again lower in high responders, presenting almost the same trend as at Day 1 (Figure 6B). Nevertheless, no statistically significant difference was observed between the two groups 3 months after the vaccination, implying alteration of plasma ceramides levels over time. The *p*-values were equal to 0.216, 0.075, 0.685 and 0.104 for Cer (D18:0/22:0), Cer (D18:1/23:0), Cer (D18:0/20:0) and Cer (D18:1/25:0), respectively.

**Figure 7:**
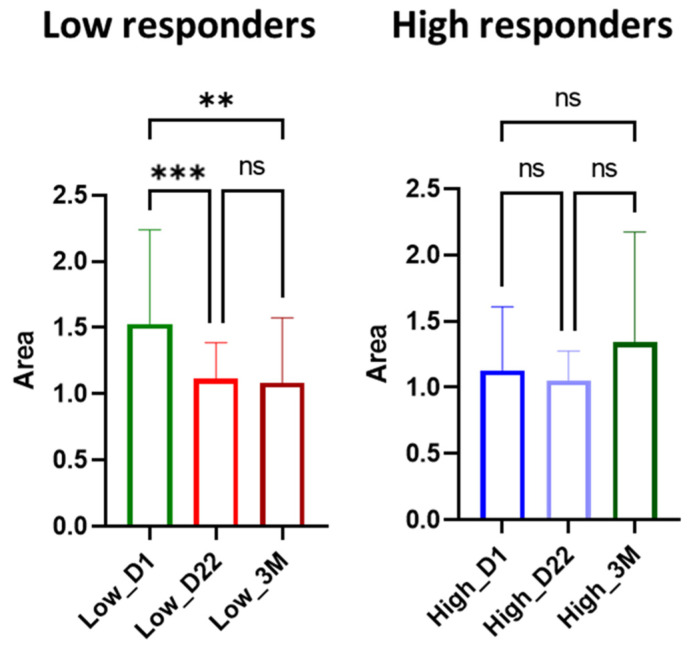
Comparison of the Cer(d18:0/22:0) levels, employing ANOVA test, among the three time points; Day 1 (D1), Day 22 (D22) and 3 Months (3M) for low and high responders. Asterisks indicate statistical significance: *** 0.0001 < *p* < 0.001, ** 0.001 < *p* < 0.01; ns: not significant.

In order to determine whether the ceramide levels alterations were due to the vaccination, the data was analyzed separately for the two groups. Specifically, the ceramide levels were compared at the three time points (first dose at Day 1, Day 22 and 3 Months) for the low, and the corresponding levels for the high responders. For example, the results for the Cer (d18:0/22:0) are presented in Figure 7. It should be noted that all the studied ceramides followed the same trend.

According to the ANOVA tests (significance level 95%), the model of the three time points for the low responders is significant (*p* = 0.0003), indicating that the means differed, whereas the model for the high responders is non-significant (*p* = 0.0644). Tukey’s multiple comparisons tests were also performed in order to study further the means among the days. The results showed that for low responders, the ceramide levels were significantly decreased 22 days after the first dose (D1 vs. D22, *p* = 0.0006), but no further decrease was observed (D22 vs. 3M, *p* = 0.9697). It suffices to say that the decrease of ceramide levels was significant between Day 1 and after three months of the first dose (D1 vs. 3M, *p* = 0.0013). For high responders, no statistically significant alteration was observed after the vaccination (D1 vs. D22, *p* = 0.800; D22 vs. 3M, *p* = 0.052; D1 vs. 3M, *p* = 0.275). It was observed that the levels of Cer (d18:0/22:0) were reduced three months after the vaccination to the levels of the high responders.

## 4. Discussion

To the best of our knowledge the current study is the first that provides a comprehensive approach to metabolomic changes induced by vaccination with the mRNA-based BNT162b2 COVID-19 vaccine and attempts to identify a potential metabolomics-based fingerprint of response to vaccination. We observed distinct plasma metabolic profiles among healthy individuals that had a strictly defined difference in response to vaccination (i.e., response after first dose), as assessed by the assessment of the levels of neutralizing antibodies (NAbs). Importantly, we used both CPMG and LED 1H NMR spectra with information on small molecules and lipids, respectively, and an untargeted UPLC MS-based approach offering a comprehensive coverage of the plasma metabolome.

Published data for the metabolomic changes during and after COVID-19 infection have shown alterations in the levels of lipoproteins, amino acids, and glucose. Kimhofer et al., used LC-MS and NMR data to show such alterations compared to healthy controls. A prediction model of SARS-CoV-2 infection based on the metabolic profile was constructed [17], employing a similar approach concerning the instrumental arrangement. A small number of lipidomic studies have shown that alterations in the lipid metabolism, including upregulated levels of triglycerides [18,19] and ceramides [20], also predispose to severe COVID-19 infection. However, in the current study we have focused on healthy, non-infected subjects who developed differential immune responses to vaccination. 

Metabolomic studies have previously examined the role of metabolic regulation in immune response to various vaccines against bacteria (e.g., *M. tuberculosis*) and viruses (e.g., influenza, variola virus), highlighting the role of purine metabolism, glycolysis, tryptophan and other amino acid pathways among others [8]. In this study, we evaluated the plasma metabolic alterations and differences observed between high and low responders in 58 healthy individuals vaccinated with the BNT162b2 COVID-19 vaccine. Distinct metabolic profiles were observed and response to vaccination was associated with alterations in the amino acid and lipid metabolism, highlighting putative biomarkers of response. Importantly, the results were consistent between the NMR and MS approaches. Interestingly, the strongest discrimination was observed on Day 1 (prior to first dose) compared to Day 22. This indicates that there are inherent metabolic differences in the plasma of low responders, already detected before the first dose of vaccination, that have the potential to predict immune response to the specific vaccine. After the 3-month period, when most of the participants had reached a high level of immune response, these differences tend to diminish, and no significant discrimination of individuals could be observed. This indicates that the identified metabolic differences may reflect the state of immune status of the individual, and the immune system’s adaptation and response. Moreover, after vaccination an intense metabolism stage (e.g., generalized inflammation) is observed, which potentially equalizes their metabolic status. These results pave the way to more personalized vaccination protocols (i.e., people with disturbed amino acid and lipid metabolisms may be at higher need of response monitoring).

### 4.1. Amino Acid Metabolism in Immune Response

In this study, baseline levels of amino acids, mostly l-histidine, 3-MH, l-phenylalanine and l-glutamine, were positively associated with response to vaccination. Amino acids are tightly related to the immune response, as a large number of amino acids are utilized to build antibodies and cytokines [21]. Several amino acids also pose a regulatory role in inflammation through the activation of innate, adaptive, and regulatory immune responses [22].

l-Histidine has been associated with immune response to acute inflammation. It has been hypothesized that this is due to the imidazole functional group related to the scavenging of reactive oxygen species produced in cells during acute inflammatory responses [23]. Involvement of l-histidine in immune response is also mediated through the production of histamine. In fact, free histidine can be catabolized to urocanic acid to form glutamate, and histidine observed in the plasma of high responders indicates an altered histidine metabolism and a decrease in histamine supply [24]. A histamine-1 receptor antagonist, rupatadine, was proposed for COVID-19 prophylaxis [25]. Histidine-rich glycoprotein, a protein dependent on the sufficiency of dietary histidine, is involved in immune responses [26] and was elevated in survivors of severe COVID-19 infection [27]. 

l-Phenylalanine is linked to immune activation as shown by increased serum levels in sepsis and HIV-infection; however, the underlying mechanisms remain unknown. Both l-phenylalanine and l-tyrosine are precursors of catecholamines, which are epinephrine-like neurotransmitters that can also act on the immune system [28,29]. Increased serum l-phenylalanine and l-tyrosine levels were observed in COVID-19 patients compared to healthy controls [13,30] and were altered along the different phases of disease [31]. l-Phenylalanine was also proposed as a biomarker of COVID-19 severity [32]. Increased phenylalanine levels in both severe COVID-19 and high response to vaccination may reflect the common immune signatures between vaccine immunization and SARS-CoV2 infection, including humoral immune response and complement activation. Moreover, vaccination with a viral vector-based vaccine against COVID-19 led to alterations in l-phenylalanine and l-tyrosine metabolism [14].

l-Glutamine is the most abundant amino acid in human plasma and is involved in several core biochemical processes, including the synthesis of proteins, nucleotides and sugars. Cells of the immune system utilize glutamine as a fuel for their proliferation and growth. Rapid division of lymphocytes is critical for efficient immune responses [33], and to do so they use glutamine as a nitrogen donor for the synthesis of nucleotides and energy production [34]. Glutamine also affects the production of cytokines by monocytes and macrophages [35] and is essential for the production of antibodies. When subjected to an inflammatory stimulus, immune cells increase the utilization and uptake of glutamine. Due to the beneficial effects of glutamine in immune responses, interventions with glutamine supplements have been examined in relation to COVID-19. Glutamine supplementation reduced the hospitalization period of COVID-19 patients [36], reduced serum levels of interleukin-1 β, hs-CRP and tumor necrosis factor-α, and increased appetite [37]. Inclusion of glutamine as an adjuvant in the treatment of COVID-19 patients showed a putative synergistic effect on immune defense [37]. Glutamine deficiency and increased hyaluran in the lungs have emerged as central metabolic characteristics that prompt COVID-19 patients to severe pathophysiology [38]. Metabolomic studies have also shown a reduced glutamine/glutamate ratio in COVID-19 patients, indicating a metabolic rewiring of energy metabolism and systematic implications during infection that could be clinically valuable [17,39]. However, since glutamine is also utilized by SARS-CoV-2 for replication, caution should be given in glutamine supplementation during infection [40]. 

### 4.2. Lipoproteins and Lipids 

Lipids are very important as key components of cellular membranes and sources or stores of energy. Several lipids are also involved in cell signaling and can modulate immune response and inflammation, including the synthesis of anti-inflammatory cytokines [41], macrophage differentiation [42] and immune cell activation [43]. On the other hand, they can induce the synthesis of pro-inflammatory cytokines, as well as promote viral entry into cells [12]. Dyslipidemia has been described as a risk factor for autoimmune and inflammatory diseases [44,45], while perturbed lipoprotein levels in relation to COVID-19 were reported by several researchers since its outbreak. Decreased total cholesterol, LDL and HDL correlated with disease severity and were proposed as predictors for the prognosis of COVID-19 patients [46,47,48,49]. Low apolipoprotein-1 and high triglyceride levels were also associated with disease severity [48,50]. Using an NMR metabolomics approach, COVID-19 patients were characterized by alterations in several lipoproteins (decreased total and HDL apolipoprotein A1, low HDL triglycerides, and increased LDL and VLDL triglycerides) using quantitative measurements of lipoprotein subfractions [17]. Cholesterol was shown to promote the interaction of spike protein with the ACE2 receptor and facilitate the entry of the virus into cells [51,52]. In this study, NMR resonances attributed to polyunsaturated fatty acids, glycerophospholipids and triacylglycerols from lipoproteins discriminated between low and high responders already before the first vaccine dose. Thus, apart from promoting viral infectivity during SARS-CoV-2 infection, lipids and lipoproteins could affect and predict vaccine-induced immune responses. 

### 4.3. Role of Ceramides in SARS-CoV-2 Antibody Production

Four of the six identified compounds by the non-targeted UPLC-MS metabolomics approach belong to the ceramide class. Ceramides are lipids composed of sphingosine and an amide-linked fatty acid chain, varying in length within the range C14:0–C26:0 making up sphingomyelin, one of the major lipids in the lipid bilayer. Sphingomyelin is cleaved into ceramides by sphingomyelinase (ASM) following infection with SARS-CoV-2, with these ceramide platforms serving as a gateway for cell infection by the virus. 

Ceramides are critically involved in immune system function [53]. Ceramide levels in human dendritic cells (DCs) are tightly regulated and their accumulation, due to inhibition of their catabolism, sensitizes DCs to ceramide-induced cell death [54]. DCs are professional antigen-presenting cells [55,56] and key players in innate and adaptive immune responses against viral infections; as expected, their involvement in development of immune response against SARS-CoV-2 infection is critical [57]. After vaccination, the mRNA contained in the vaccine enters DCs at the injection site or within lymph nodes and high levels of the viral S protein are produced. The DCs are activated and present the antigen (S protein) to S protein-specific naive T cells, which are differentiated and form cytotoxic T lymphocytes or helper T cells. Subsequently, B cells are differentiated into antibody-secreting plasma cells and high affinity anti-S protein antibodies are produced. After the mRNA vaccination, S protein-specific memory T and B cells as well as SARS-CoV-2 antibodies circulate, preventing a subsequent infection [58,59]. Thus, DCs have a key role in SARS-CoV-2 antibody production and their levels and functional capacity could be critical to the level of immune activation and response. Accumulation of ceramides leads to DC death, and inhibits the antigen-capturing ability and presentation of DCs [60]; thus in individuals with high levels of ceramides, a “blunted” DC response could partially explain the lower levels of antibodies 22 days after the first vaccine dose.

### 4.4. Strengths and Limitations

The main advantage of this study was the combined use of the two main analytical techniques exploited in metabolomics, NMR and MS, which offered a comprehensive coverage of the plasma metabolome. With untargeted NMR approaches up to one or a few hundreds of metabolites can be detected in a single run due to overlapping signals, compared to thousands of metabolites that can be detected in MS applications due to higher sensitivity. However, NMR results are very reproducible, and the confidence level of annotation is high. On the other hand, MS-based approaches have higher sensitivity and provide a wider range of metabolites with structure identification afforded by MS^n^ analysis. In our analysis, LED NMR experiments highlighted the effect of lipids in immune responses; however, these signals only provided information on the chemical moieties present in several lipid classes and lipoproteins rather than a single biomarker. Our MS analysis was able to provide further information regarding the affected lipid classes and suggested plasma ceramides as biomarkers of response. Fragment fingerprinting using MS^n^ experiments also led to high confidence of assignment. On the other hand, NMR provided robust and reproducible information regarding amino acid levels, further exploiting the “SMolESY-select” platform for targeted NMR metabolite identification. Finally, the associated metabolites were examined in three core time points of vaccination, providing new insights on time-dependent metabolic alterations.

A limitation of our study was the relatively small size of the cohort. Considering the variability within the low/high responders’ groups regarding gender, age, smokers/nonsmokers and other lifestyle factors, subsequent studies with larger sample size may be required to overcome within-group variability issues; however, our analyses were corrected for age and gender, which are known to be implicated in vaccine-induced immune responses. The limited size did not allow adjustment for other lifestyle factors; nonetheless, all the subjects were “healthy” without any known immunosuppression. However, it is notable that the discrimination of individuals was based on the evaluation of NAb levels, which provides a more reliable (and validated) evaluation of the humoral immune response that is not restricted to the measurement of specific immunoglobulin subclasses. In the current study, we have not replicated the results in independent cohorts, wherein the significantly identified metabolites (or the candidate metabolic fingerprint) should be further validated. Finally, further experimental work with in vitro and/or in vivo studies are required for a causal relationship between amino acid metabolism and ceramides in response to BNT162b2 COVID-19 vaccination to be established. 

## 5. Conclusions

In summary, distinct plasma metabolic profiles were observed in healthy individuals who had differential immune responses after the first dose of the mRNA BNT162b2 COVID-19 vaccine. Our analysis highlighted the role of amino acid metabolism and the lipid profile as predictive markers of response to vaccination while the levels of plasma ceramides indicate that this class of lipids, along with certain amino acids, could emerge as a predictive biomarker for immune response and severity of inflammation. Moreover, these findings could drive the search for ways to reduce levels of ceramides via the use of ASM inhibitors. 

## Figures and Tables

**Table 1:** Demographic characteristics and number of antibodies for the participants enrolled in this study for each of the “Low” and “High” response groups.

	High Responders	Low Responders
No. Observations	29	29
Gender		
Female	23	17
Male	6	12
Age		
Minimum	27	25
Maximum	64	68
1st Quartile	33	51
Median	45	58
3rd Quartile	54	64
Mean	43.79	54.86
Standard deviation	11.99	12.15
NAbs titers, Day 1		
Minimum	0	0
Maximum	43.753	25.64
1st Quartile	5.363	4.142
Median	14.607	9.076
3rd Quartile	23.955	12.762
Mean	14.888	9.55
Standard deviation	11.474	6.916
NAbs titers, Day 22		
Minimum	72.027	6.971
Maximum	98.23	38.146
1st Quartile	76.894	22.859
Median	81.742	30.383
3rd Quartile	86.179	34.703
Mean	82.9	27.531
Standard deviation	7.412	8.663
NAbs titers, 3 Months		
Minimum	78.478	41.33
Maximum	98.036	96.997
1st Quartile	93.266	78.567
Median	96.682	84.074
3rd Quartile	97.328	91.331
Mean	94.349	82.04
Standard deviation	4.9	13.909

**Table 2:** Metabolites associated with response at first dose at FDR 95% confidence level (*p*-value < 2.3 × 10^−4^). The partial Spearman correlation between response and each NMR feature was adjusted for age and sex. Significant spectral areas that were assigned to a known metabolite along with the partial correlation coefficient (r) and smallest associated *p*-value is shown. Index is the numbering used in Figure 1. Positive r indicates direct association with high response at first dose.

Metabolite	Peak ppm Range	Index	ppm with Smallest *p*-Value	*p*-Value	Partial Correlation r
l-Histidine	[7.739–7.812]	3	7.772	2.49 × 10^−6^	0.5824
l-Histidine	[7.041–7.069]	3	7.052	1.71 × 10^−6^	0.5899
l-Phenylalanine	[7.400–7.446]	5	7.422	6.03 × 10^−5^	0.5096
l-Phenylalanine	[7.306–7.345]	5	7.333	1.12 × 10^−4^	0.4933
l-Phenylalanine	[7.359–7.387]	5	7.38	1.56 ×1 0^−4^	0.4842
l-Histidine; l-Phenylalanine	Overlapping area	3/5	3.955	1.67 × 10^−5^	0.5410
3-Methylhistidine	[7.649–7.677]	4	7.653	4.40 × 10^−4^	0.4541
3-Methylhistidine	[6.944–6.996]	4	6.951	5.84 × 10^−5^	0.5105
l-Glutamine	[2.092–2.153] *	14	2.136	2.66 × 10^−5^	0.5300
l-Glutamine	[2.428–2.470]	14	2.431	8.60 × 10^−5^	0.5004

* ppm range overlapping with l-glutamic acid.

**Table 3:** Candidate metabolites identified in low and high responders from the plasma-based metabolomic approach obtained from both (+) and (−) ESI analyses. The features are presented as a. *m*/*z* feature, b. t_R_ (min), c. theoretical *m*/*z*, d. Δm (ppm), e. possible identification name, f. possible molecular formula, g. corresponding monoisotopic exact mass, h. trend in low responders (up or down regulation), i. dataset (positive or negative ESI mode) and j. adducts.

*m*/*z*	t_R_ (min)	Theoretical *m*/*z*	Δm (ppm)	Name	Molecular Formula	Exact Mass	Trend ^a^	Dataset	Adduct
622.6114	18.92	622.6133	−3.05	Cer(D18:0/22:0)	C_40_H_81_NO_3_	623.6211	↑	ESI (−)	-
646.6098	18.81	646.6109	−1.70					ESI (+)	[M+Na]
634.6120	18.99	634.6133	−2.05	Cer(D18:1/23:0)	C_41_H_81_NO_3_	635.6211	↑	ESI (−)	-
658.6120	18.96	658.6109	1.67					ESI (+)	[M+Na]
594.5802	18.28	594.5820	−3.02	Cer(D18:0/20:0)	C_28_H_77_NO_3_	595.5898	↑	ESI (−)	-
684.6241	19.39	684.6265	−3.49	Cer(D18:1/25:0)	C_43_H_85_NO_3_	663.6524	↑	ESI (−)	[M+Na-2H]
436.2816	15.30	436.2823	−1.60	LysoPE(P-16:0/0:0)	C_21_H_44_NO_6_P	437.2901	↑	ESI (−)	-
552.3055	14.65	552.3085	−5.33	LysoPE(0:0/24:6) or LysoPE(24:6/0:0)	C_29_H_48_NO_7_P	553.3163	↑	ESI (−)	-

^a^: (↑): up regulated in low responders.

## Data Availability

The data presented in this study are available in this article and supplementary material.

## Supplementary Materials

The following supporting information can be downloaded at: https://www.mdpi.com/article/10.3390/cells11071241/s1, Table S1: Detailed NAbs measurements of all individuals at Day 1, Day 22 and 3 months after first vaccination. Table S2: Chemical shifts of annotated metabolites and respective multiplicity of peaks. s: singlet; d: doublet; t: triplet; q: quadruplet and dd: doublet of doublets; Table S3: Detailed CPMG spectral bins associated with response at first dose at FDR 95% confidence level (*p*-value < 2.29 × 10^−4^ respectively). The partial Spearman correlation coefficients of each significant spectral bin along with their *p*-values are shown. Analysis was adjusted for age and sex. Assignment of spectral bins to known metabolites are shown; Table S4: Spectral variables and assigned metabolites contributing the most to the discrimination of high and low responders at Day 1 as derived from the S-plot from the orthogonal partial least squares- discriminant analysis of NMR CPMG from plasma samples shown in Figure 4. The table shows the number of significant spectral bins attributed to the same metabolite (# bins), as well as the overlapping metabolites when peak overlay occurs; Table S5: Information on lipid annotation along with Fold Change (FC) values (average levels in the low responders/average levels in the high responders) and *p*-values from *t*-test. FC free cholesterol, CE cholesterol ester, STOCSY statistical correlation spectroscopy, TG triacylglycerols, GPL glycerophospholipids, PUFA polyunsaturated fatty acids, MUFA monounsaturated fatty acids, and LPC lysophosphocholines; Figure S1: (A) Aromatic (δ 6.6–8.5), (B) aliphatic (δ 3.3–4.3; the EDTA region is not shown), and (C) aliphatic (δ 0.8–2.4) regions of the 58 spectra (cyan) and their mean (red) (upper panels) along with corresponding Manhattan-type plot showing the analysis of the 6555 CPMG NMR features with response as low or high levels of NAbs (lower panels). The signed −log10(*p*-value) is derived from Spearman correlation analysis with adjustment for age and sex. The dotted lines represent the threshold after multiple Bonferroni testing correction (blue line, *p*-value < 1.8 × 10^−5^) or FDR (black, *p*-value < 2.3× 10^−4^) and the red dots represent the data points that remain significant after FDR. The horizontal axis is the NMR chemical shift (δ, ppm). Histidines, phenylalanine and glutamine levels at Day 1 are positively associated with response. 1: Formic acid, 2: Hypoxanthine, 3: l-Histidine, 4: 3-Methylhistidine, 5. l-Phenylalanine, 6. l-Tyrosine, 7. l-Threonine, 8. l-Proline, 9. Lactic acid, 10. Creatinine, 11. Unknown, 12. d-Glucose, 13. l-Alanine, 14. l-Glutamine, 15. l-Glutamic acid, 16. Glycine, 17. Acetoacetic acid, 18. Methanol, 19. Proline betaine, 20. Succinic acid, 21. Pyruvic acid, 22. Acetone, 23. Acetic acid, 24. l-Lysine, 25. l-Leucine, 26. 3-Hydroxyisobutyric acid, 27. l-Valine, 28. l-Isoleucine; Figure S2: NMR pseudo-spectrum showing the covariance between the 6555 CPMG NMR features in the spectra of Day 1 and immune response (low vs. high NAb levels). Coloring based on the correlation coefficient, with red indicating a strong positive correlation and blue a strong negative correlation. (A) Aromatic (δ 8.50–5.80), (B) aliphatic (δ 4.40–3.25) and (C) aliphatic (δ 2.50–0.70) regions. The EDTA region has been removed. The horizontal axis is the NMR chemical shift (δ, ppm). Numbering is referring to annotation shown in Figure S1. Peaks denoted with L are lipids described in Table S5 (LED spectra); Figure S3: Fold-change of high vs. low responders of metabolic ratios in (A) Day 22, (B) 3 Months. The changes in the metabolic ratios that were statistically significant using *t*-test (*p*-value < 0.05) are in boxes. Color coding is based on conditional formatting of fold-change values; Figure S4: Scores plots obtained from the PCA of NMR CPMG spectra of plasma samples from high vs. low responders at (A) Day 1, (B) Day 22, and (C) 3 Months. It is observed that a trend for discrimination is already observed from Day 1, while this trend is diminished at 3 months.; Figure S5: Permutation testing results from the OPLS-DA of NMR CPMG spectra from plasma samples of high vs. low responders at (A) Day 1, (B) Day 22 and (C) 3 Months. In order to assess the validity of the generated classifications, 100 random permutations were performed, and the quality parameters are plotted (on the left) along/together with R2X and Q2 of the original model (on the right). Validity of the Day 1 and Day 22 models was proven (“random” values lower than model parameters); Figure S6: OPLS-DA of NMR CPMG from plasma samples of female high vs. low responders at Day 1. (A) Scores plot. The two groups are discriminated. (B) S-plot. Spectral variables on the top right corner (blue) are considered significantly increased in the high responders, while those on the bottom left corner (red) are increased in the low responders. Metabolites with *p* and *p(corr*)~0 (coloured in grey) do not significantly impact the separation. Significant spectral variables and their assigned metabolites are shown. (C) Permutation testing results (100 random permutations); Figure S7: OPLS-DA of NMR CPMG from plasmasamples of male high vs. low responders at Day 1. (A) Scores plot. The two groups are discriminated. (B) S-plot. Spectral variables on the up- right corner (blue) are considered significantly increased in the high responders, while those on the down-left corner (red) are increased in the low responders. Metabolites with *p* and *p(corr)*~0 (coloured in grey) do not significantly impact the separation. Significant spectral variables and their assigned metabolites are shown. (C) Permutation testing results (100 random permutations); Figure S8: Permutation testing results (100 random permutations) from the OPLS-DA of NMR LED spectra; Figure S9: STOCSY plot using (A) δ = 0.68 (increased in the high responders) and (B) δ = 5.21 (increased in the low responders) as “driver peaks” [shown with an arrow]. Signal assignments were based on the fact that spectral features exhibiting high correlation belong on the same molecule or lipid class. Numbering of lipids is the same as in Figure 4A. (C) Loadings plot (in line format) from the OPLS-DA of NMR LED spectra from plasma samples of high vs. low responders after first dose. Spectral variables increased in the high responders (red) and increased in the low responders group (blue). Coloring based on the correlation *p*(corr). The pq1 value refers to the weight that combines the X and Y loadings (*p* and *q*); Figure S10: ROC curves of the annotated features; Figure S11: Box and whiskers plots of the detected ceramides for male vs. female at Day 1. The differences were not statistically significant; *p*-values > 0.05. ns: not significant.
