# Supplementary material for: Plasma Metabolomic Alterations Induced by COVID-19 Vaccination Reveal Putative Biomarkers Reflecting the Immune Response
Source: Cells. 2022 Apr 6;11(7):1241. doi: 10.3390/cells11071241 (PMC8997405; doi:10.3390/cells11071241)
Supplement: Supplementary file 1 [file cells-11-01241-s001.zip › cells-1629654-supplementary.pdf]

Supplementary

Table of Contents

Supplementary S1. ... 2

1 Tables ... 2

2 Figures ... 7

Supplementary S2. ... 16

3 Materials and Methods ... 16

3.1 Chemicals and Reagents ... 16

3.2 Sample preparation ... 16

3.2.1 NMR ... 16

3.2.2 MS ... 16

3.3 Data Acquisition ... 17

3.3.1 NMR Data Acquisition ... 17

3.3.2 LC – MS Data Acquisition ... 17

3.4 Data Processing ... 19

3.4.1 NMR Processing ... 19

3.4.2 LC – MS Processing ... 21

3.4.3 NMR and LC – MS Multivariate Statistical Analysis ... 28

Supplementary S1.

1 Tables

Table S1: Detailed NAbs measurements of all individuals at Day 1, Day 22 and 3 months after first vaccination.

Sample	D1	D22	3M
Low_1	0.00	18.21	95.12
Low_2	25.64	35.73	71.72
Low_3	5.38	33.18	91.33
Low_4	4.76	14.50	96.30
Low_5	22.40	25.30	81.32
Low_6	2.69	31.20	90.09
Low_7	3.14	38.15	68.18
Low_8	12.76	22.86	96.60
Low_9	11.96	24.41	91.16
Low_10	5.26	29.33	84.07
Low_11	3.55	34.70	96.49
Low_12	13.82	6.97	46.21
Low_13	19.99	25.41	68.66
Low_14	11.71	12.19	80.12
Low_15	5.08	17.88	84.76
Low_16	3.67	30.38	83.27
Low_17	11.85	23.65	85.64
Low_18	15.67	35.53	41.33
Low_19	11.39	36.40	78.57
Low_20	7.00	17.37	66.01
Low_21	4.14	30.15	83.82
Low_22	0.00	37.91	86.09
Low_23	0.00	33.81	78.94
Low_24	14.61	15.96	97.00
Low_25	11.17	31.29	75.15
Low_26	21.49	37.26	82.27
Low_27	8.12	36.59	94.86
Low_28	9.08	31.10	90.48
Low_29	10.64	31.00	93.59

Sample	D1	D22	3M
High_1	4.76	72.67	84.38
High_2	25.05	82.69	97.55
High_3	12.22	95.41	97.67
High_4	10.77	83.91	98.04
High_5	14.61	76.53	86.45
High_6	10.41	91.84	97.70
High_7	15.53	89.10	97.25
High_8	8.07	72.03	91.55
High_9	24.43	81.74	93.62
High_10	20.55	85.85	96.68
High_11	25.52	80.83	97.33
High_12	23.95	98.23	93.27
High_13	30.11	80.69	95.30
High_14	21.79	76.89	96.96
High_15	1.96	78.94	97.12
High_16	4.41	81.88	95.88
High_17	6.91	84.35	95.71
High_18	27.01	76.44	92.47
High_19	5.36	74.89	96.74
High_20	0.00	89.33	97.02
High_21	15.49	94.33	95.10
High_22	5.98	81.70	97.45
High_23	15.18	73.65	84.38
High_24	43.75	85.73	96.51
High_25	0.00	86.18	97.40
High_26	0.00	77.81	97.28
High_27	2.54	75.91	78.48
High_28	18.89	77.45	93.22
High_29	36.49	97.10	97.64

Table S2: Chemical shifts of annotated metabolites and respective multiplicity of peaks. s: singlet; d: doublet; t: triplet; q: quadruplet and dd: doublet of doublets.

Metabolites	1H δ (ppm) - Multiplicity
(S)-3-Hydroxyisobutyric acid	1.06 d
3-Hydroxybutyric acid	1.19 d
3-Methylhistidine	7.65 s; 6.95 s
Acetic acid	1.91 s
Acetoacetic acid	3.43 s
Acetone	2.22 s
Alpha-ketoisovaleric acid	1.11 d
Choline	4.06 m; 3.19 s
cis-Aconitic acid	5.68 d;
Creatine	3.95 s; 3.03 s
Creatinine	4.04s; 3.03 s
D-Glucose	5.23 d; 4.64 d; 3.89 dd; 3.83 m; 3.75 q; 3.72 dd; 3.71 t; 3.53 dd; 3.49 t; 3.45 m; 3.41 m; 3.24 m
Dimethyl sulfone	3.14 s
D-Mannose	5.18 d
Ethanol	1.18 t
Formic acid	8.45 s
Glycerol	3.65 m; 3.56 m
Hypoxanthine	8.18 s
Isopropyl alcohol	1.16 d
L-Alanine	3.78 q; 1.47 d
L-Carnitine	3.22 s
L-Glutamic acid	2.35 m; 3.74 m; 2.14 m; 2.05 m
L-Glutamine	3.77 t; 2.48 m; 2.45 m; 2.14 m; 2.12 m
Glycine	3.55 s
L-Histidine	7.77 d, 7.05 s, 3.98 q
L-Isoleucine	1.00 d; 0.93 t
L-Lactic acid	4.10 q; 1.32 d
L-Leucine	3.74 q; 1.72 m; 0.97 d; 0.96 d
L-Lysine	1.90 m; 1.72 m; 3.02 m
L-Phenylalanine	7.42 t; 7.37 t; 7.32 d; 3.98 dd
L-Proline	3.34 m; 4.12 dd; 2.34 m; 2.06 m; 2.02 m; 1.98 m
L-Threonine	4.24 m; 3.57 d; 1.33 d
L-Tyrosine	7.19 d; 6.89 d
L-Valine	1.03 d; 0.98 d; 3.60 d; 2.26 m
Methanol	3.36 s
Myo-inositol	4.06 t; 3.30 t
Ornithine	3.05 t; 3.78 t; 1.92 m; 1.82 m; 1.74 m
Proline betaine	3.29 s
Propylene glycol	1.14 d
Pyruvic acid	2.36 s
Succinic acid	2.41 s

Table S3: Detailed CPMG spectral bins associated with response at 1st dose at FDR 95% confidence level (*p*-value<2.29 x 10^{-4} respectively). The partial Spearman correlation coefficients of each

significant spectral bin along with their *p*-values are shown. Analysis was adjusted for age and sex. Assignment of spectral bins to known metabolites are shown.

(δ) ppm	Metabolite	*p*-value	Partial correlation coefficient
8.281		4.58E-05	-0.5166
7.88		6.97E-05	-0.5059
7.774	L-Histidine	2.10E-04	0.4758
7.773	L-Histidine	9.25E-06	0.5545
7.772	L-Histidine	2.49E-06	0.5824
7.771	L-Histidine	1.15E-04	0.4925
7.435	L-Phenylalanine	9.15E-05	0.4987
7.434	L-Phenylalanine	2.05E-04	0.4766
7.422	L-Phenylalanine	6.03E-05	0.5096
7.38	L-Phenylalanine	1.56E-04	0.4842
7.333	L-Phenylalanine	1.12E-04	0.4933
7.332	L-Phenylalanine	2.29E-04	0.4734
7.054	L-Histidine	1.42E-04	0.4869
7.053	L-Histidine	1.31E-05	0.5466
7.052	L-Histidine	1.71E-06	0.5899
7.051	L-Histidine	6.35E-05	0.5083
6.951	3-Methylhistidine	5.84E-05	0.5105
6.949	3-Methylhistidine	8.09E-05	0.502
6.745		5.10E-05	-0.5139
4.237	L-Threonine; glyceryl moiety	8.07E-05	-0.5021
4.236	L-Threonine; glyceryl moiety	1.35E-04	-0.4883
3.955	L-Histidine; L-Tyrosine; L-Phenylalanine	1.67E-05	0.541
2.431	L-Glutamine	8.60E-05	0.5004
2.16	L-Glutamine	1.56E-04	0.4842
2.146	L-Glutamine	1.36E-04	0.4881
2.137	L-Glutamine	2.15E-04	0.4752
2.136	L-Glutamine	2.66E-05	0.53
2.135	L-Glutamine	3.20E-05	0.5255
2.125	L-Glutamine; L-Glutamic acid	1.18E-04	0.4919
2.124	L-Glutamine; L-Glutamic acid	3.09E-05	0.5264
2.111	L-Glutamine; L-Glutamic acid	4.48E-05	0.5172
2.11	L-Glutamine; L-Glutamic acid	6.04E-05	0.5096
1.155		0.000165	0.4827

Table S4: Spectral variables and assigned metabolites contributing the most to the discrimination of high and low responders at Day 1 as derived from the S-plot from the orthogonal partial least squares-discriminant analysis of NMR CPMG from plasma samples shown in Figure 4. The table shows the number of significant spectral bins that have been attributed to the same metabolite (# bins), as well as the overlapping metabolite when peak overlay occurs.

	**Increased in low responders**	
**(δ) ppm**	**# bins**	**Associated metabolite**
2.22	4	Acetone; C<u>H</u>$_2$CO (fatty acyl chains)
1.25-1.33	81	CH2 from lipoproteins; CH3-**CH2**(CH2)n,(**CH2**)n (fatty acyl chains)
1.037, 1.025	2	L-Valine; (CH2)n (fatty acyl chains)
0.986	1	L-Valine; CH3 (C19 from Cholesterol)
0.86-0.88	25	CH3 from lipoproteins; **CH3**-CH2-C=, **CH3**-CH2CH2C= (fatty acyl chains)
	**Increased in high responders**	
**(δ) ppm**	**# bins**	**Associated metabolite**
7.77, 7.05	6	L-Histidine
7.195, 6.9	2	L-Tyrosine
3.94-3.97	10	L-Histidine; L-Tyrosine; L-Phenylalanine
3.887, 3.886	2	D-Glucose
3.74-3.75, 3.763	9	L-Alanine; L-Lysine
3.565, 3.566	2	Glycerol
3.55	6	Glycine
3.052	1	L-Ornithine; L-Tyrosine
3.03	2	Creatinine
3.02	3	L-Lysine
2.43-2.47	19	L-Glutamine
2.409, 2.41	2	Succinic acid
2.22	3	Acetone; C<u>H</u>$_2$CO (fatty acyl chains)
2.10-2.14	22	L-Glutamine (overlapping with L-Glutamic acid)
2.07	3	Unknown; L-Proline
2.03-2.04	5	CH2C=C (fatty acyl chains); L-Proline
1.47-1.48	6	L-Alanine
1.315,1.327	2	L-Lactic acid
1.21-1.24	23	CH2 from lipoproteins; CH3-**CH2**(CH2)n, (**CH2**)n (fatty acyl chains)
1.03-1.04	3	L-Valine; (CH2)n (fatty acyl chains)
0.98-0.99	4	L-Valine; CH3 (C19 from Cholesterol)
0.953	1	L-Leucine, CH3 from lipoproteins
0.82-0.85	37	**CH3**-(CH2)n; CH3 from lipoproteins

Table S5: Information on lipid annotation along with Fold Change (FC) values (Average levels in the low responders/ Average levels in the high responders) and *p*-values from t-test. FC free cholesterol, CE cholesterol ester, STOCSY statistical correlation spectroscopy, TG triacylglycerols, GPL glycerophospholipids, PUFA polyunsaturated fatty acids, MUFA monounsaturated fatty acids, LPC lysophosphocholines.

INDEX	PPM	Signals	Assignment	FC	*p*-value*
L1	0.64–0.74	Lipids CH3- (C18 cholesterol);	Cholesterol (FC, CE)	0.943	0.0018
L2	0.81–0.88	Lipids CH3- (C21 cholesterol) ; Methyl group **CH3**-(CH2)n	Cholesterol	0.932	0.0122
L3	0.89–0.92	Lipids CH3-; Methyl group **CH3**-CH2-C=, **CH3**-CH2CH2C=	Fatty acids	1.111	0.0116
L4	1.21–1.26	Lipids CH2-; Methylene group CH3-**CH2**(CH2)n, (**CH2**)n	STOCSY with cholesterol	0.953	0.1434
L5	1.27–1.39	Lipids CH2-; Methylene group CH3-**CH2**(CH2)n, (**CH2**)n	STOCSY with TG, GPL, PUFA	1.158	0.0002
L6	1.47–1.56	Lipids; Methylene group -**CH2**-CH2-CH= or -**CH2**CH2-C=O	STOCSY with cholesterol	0.951	0.0019
L7	1.574–1.633	Lipids; Methylene group -**CH2**-CH2-CH= or -**CH2**CH2-C=O	STOCSY with TG, GPL, PUFA	1.061	0.0117
L8	1.94–2.04	Methylene group next to double bond -**CH2**-CH=	MUFA/cholesterol	0.993	0.4584
L9	2.05–2.08	Methylene group next to double bond -C**H2**-CH=, N-acetylglycoproteins (NCH3-)	PUFA	1.025	0.1227
L10	2.21–2.274	Methylene group next to double bond -**CH2**-C=O	Fatty acids	1.067	0.0099
L11	3.63–3.76	C3 Cholesterol	Cholesterol	0.928	0.0014
L12	3.87–3.951	Unknown		0.934	0.0033
L13	4.308–4.225	Glyceryl group **CH2**OCOR; other molecules overlap here (from cholines)	TG, GPL (except LPC)	0.976	0.0513
L14	5.19–5.236	Glyceryl group **CH**OCOR	TG, GPL (except LPC)	1.150	0.0069
L15	5.25–5.40	Methine group **CH**=CH	Unsaturated fatty acids	1.072	0.0014

* T-test with Welch's correction and Mann-Whitney test have been applied in case of unequal variances and non-normal distributions, respectively.

2 Figures

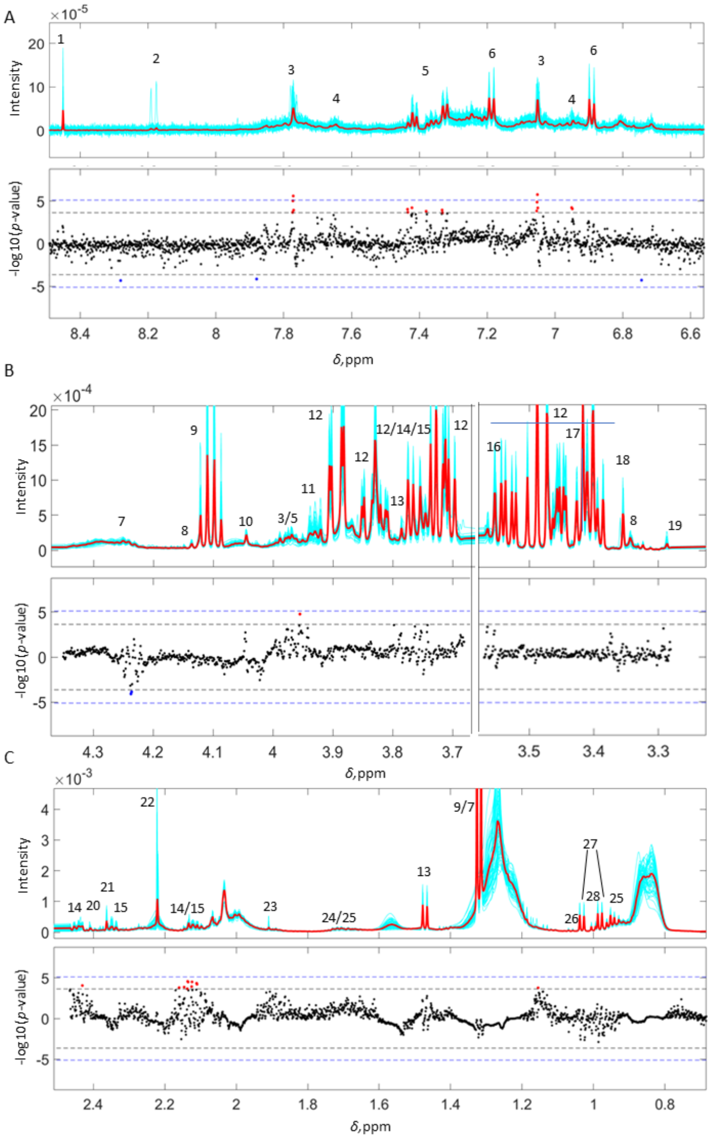

Figure S1: A) Aromatic (δ 6.6–8.5), B) Aliphatic (δ 3.3–4.3; the EDTA region is not shown), and C) Aliphatic (δ 0.8-2.4) regions of the 58 spectra (cyan) and their mean (red) (upper panels) along with corresponding Manhattan-type plot showing the analysis of the 6555 CPMG NMR features with response as low or high levels of NAbs (lower panels). The signed −log10(*p*-value) is derived from Spearman correlation analysis with adjustment for age and sex. The dotted lines represent the threshold after multiple testing correction Bonferroni (blue line, *p*-value<1.8×10^{-5}) or FDR (black, *p*-value<2.3×10^{-4}), the red dots represent the data points that remain significant after FDR. The horizontal axis is the NMR chemical shift (*δ*, ppm). Histidines, phenylalanine and glutamine levels at Day 1 are positively associated with response. 1: Formic acid, 2: Hypoxanthine, 3: L-Histidine, 4: 3-Methylhistidine, 5. L-Phenylalanine, 6. L-Tyrosine, 7. L-Threonine, 8. L-Proline, 9. Lactic acid, 10. Creatinine, 11. Unknown, 12. D-Glucose, 13. L-Alanine, 14. L-Glutamine, 15. L-Glutamic acid, 16. Glycine, 17. Acetoacetic acid, 18. Methanol, 19. Proline betaine, 20. Succinic acid, 21. Pyruvic acid, 22. Acetone, 23. Acetic acid, 24. L-Lysine, 25. L-Leucine, 26. 3-Hydroxyisobutyric acid, 27. L-Valine, 28. L-Isoleucine.

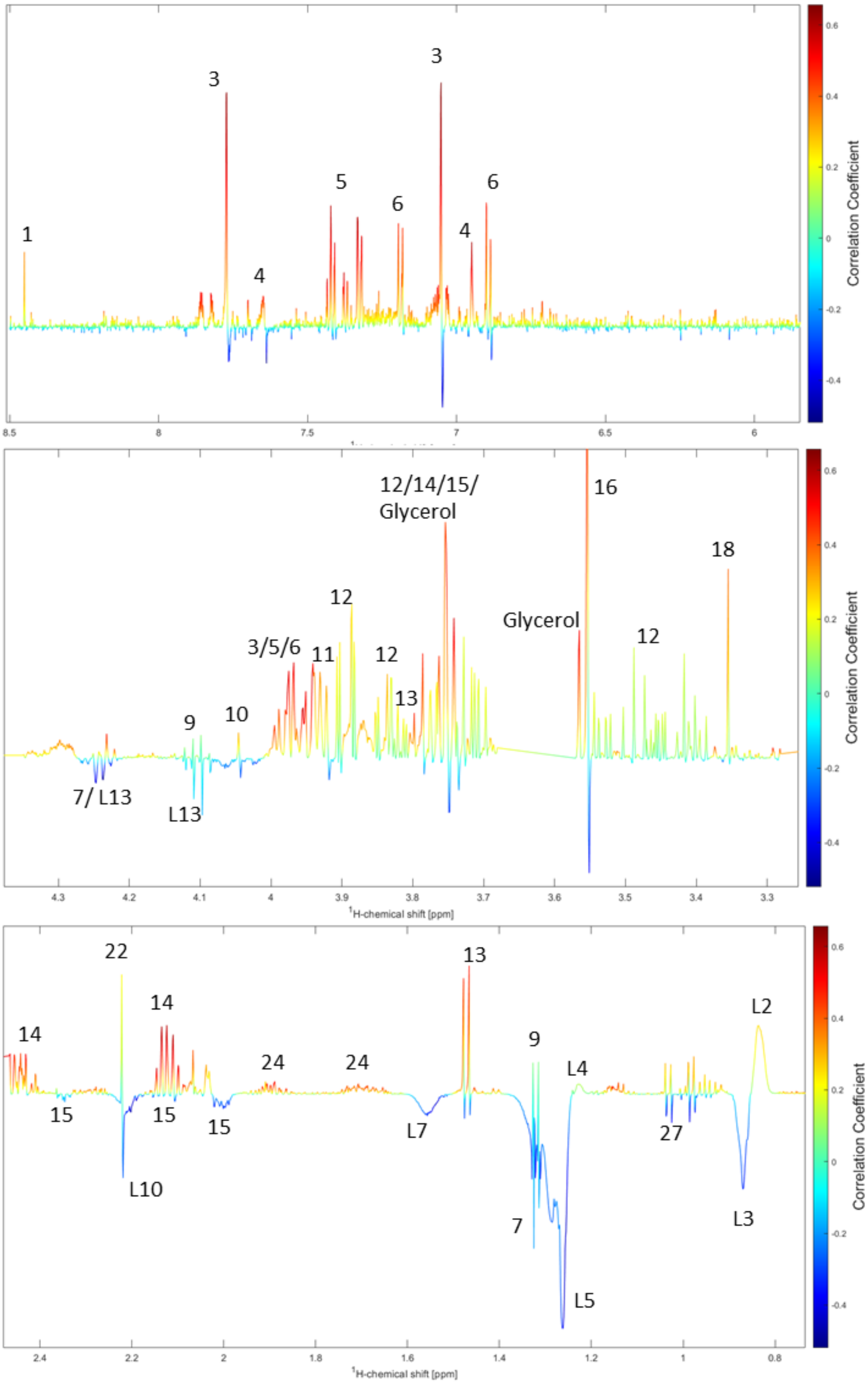

Figure S2: NMR pseudo-spectrum showing the covariance between the 6555 CPMG NMR features in the spectra of Day 1 and immune response (low vs high NAbs levels). Coloring based on the correlation coefficient, with red indicating a strong positive correlation and blue a strong negative correlation. A) Aromatic (δ 8.50–5.80), B) Aliphatic (δ 4.40–3.25) and C) Aliphatic (δ 2.50–0.70) regions. The EDTA region has been removed. The horizontal axis is the NMR chemical shift (δ, ppm). Numbering is referring to annotation shown in Supplementary Figure 1. Peaks denoted with L are lipids described in Supplementary Table A4 (LED spectra).

A

Numerator

Denominator	3-Hydroxybutyric acid	Acetic acid	Acetone	Choline	Creatine	Creatinine	D-Glucose	Dimethyl sulfone	Formic acid	Glycine	Lactic acid	L-Alanine	L-Glutamine	L-Histidine	L-Isoleucine	L-Leucine	L-Lysine	L-Phenylalanine	L-Tyrosine	L-Valine
3-Hydroxybutyric acid	1.00	1.16	2.24	1.48	1.31	0.99	0.95	0.87	1.08	1.07	1.13	0.77	0.98	1.18	1.33	1.23	1.08	1.41	1.13	1.16
Acetic acid	0.86	1.00	4.24	1.05	1.01	0.89	0.89	0.94	0.89	1.09	0.97	0.99	1.20	1.40	0.94	0.95	1.33	1.00	0.88	0.81
Acetone	0.45	0.24	1.00	0.23	0.51	0.12	0.14	0.20	0.65	0.14	0.12	0.13	0.19	0.21	0.16	0.28	0.31	0.20	0.19	0.14
Choline	0.68	0.97	4.41	1.00	1.23	0.91	0.80	0.81	0.89	1.02	0.77	0.87	0.94	0.95	0.82	0.99	1.28	0.97	0.93	0.81
Creatine	0.76	0.99	1.95	0.84	1.00	0.90	0.86	0.74	0.68	1.01	0.66	0.88	1.07	0.95	0.76	0.82	1.08	1.27	0.92	0.76
Creatinine	1.01	1.12	8.09	1.14	1.11	1.00	0.99	0.95	0.79	1.06	0.88	0.91	1.11	1.32	0.92	1.05	1.04	1.15	0.91	1.01
D-Glucose	1.05	1.12	7.24	1.30	1.17	1.01	1.00	0.75	0.94	1.13	1.00	1.03	1.19	1.26	0.99	1.03	1.44	1.09	1.02	1.02
Dimethyl sulfone	1.15	1.06	5.04	1.24	1.36	1.05	1.34	1.00	1.00	1.22	0.93	1.07	1.22	1.49	1.17	1.07	1.50	1.37	1.22	1.13
Formic acid	0.93	1.13	1.55	1.14	1.46	1.27	1.06	1.00	1.00	1.09	0.73	1.07	1.31	1.22	1.11	1.28	1.27	1.33	0.97	1.13
Glycine	0.94	0.91	7.01	1.01	0.99	0.95	0.88	0.82	0.92	1.00	0.71	0.76	1.01	1.16	0.79	0.81	1.13	0.95	0.82	0.87
Lactic acid	0.89	1.03	8.30	1.31	1.53	1.14	1.00	1.07	1.38	1.41	1.00	1.20	1.33	1.38	1.11	1.31	1.68	1.33	1.19	1.12
L-Alanine	1.31	1.01	7.48	1.18	1.13	1.09	0.97	0.94	0.94	1.31	0.83	1.00	1.10	1.23	0.99	1.01	1.21	1.16	1.06	0.95
L-Glutamine	0.87	0.87	5.80	1.14	1.04	0.97	0.81	0.84	0.82	1.07	0.79	0.95	1.00	1.05	0.87	0.92	0.99	0.89	0.89	0.82
L-Histidine	0.85	0.72	4.70	1.09	1.06	0.76	0.80	0.67	0.82	0.86	0.72	0.81	1.01	1.00	0.77	0.91	0.94	0.97	0.86	0.79
L-Isoleucine	0.75	1.06	6.40	1.30	1.32	1.09	1.01	0.86	0.90	1.27	0.90	1.01	1.31	1.30	1.00	1.05	1.56	1.13	0.97	0.99
L-Leucine	0.82	1.05	6.40	1.30	1.22	0.95	0.98	0.93	0.78	1.23	0.76	0.99	1.16	1.09	0.95	1.00	1.31	1.16	0.94	1.01
L-Lysine	0.93	0.75	3.28	0.81	0.92	0.96	0.69	0.67	0.79	0.89	0.60	0.83	1.03	1.06	0.64	0.76	1.00	0.99	0.78	0.72
L-Phenylalanine	0.71	1.00	4.99	1.04	0.79	0.87	0.92	0.73	0.75	1.05	0.75	0.86	1.14	1.04	0.89	0.86	1.01	1.00	0.95	0.84
L-Tyrosine	0.88	1.14	5.40	1.08	1.09	1.10	0.98	0.82	1.03	1.22	0.84	0.94	1.32	1.16	1.03	1.07	1.28	1.05	1.00	1.06
L-Valine	0.86	1.24	6.93	1.28	1.32	0.99	0.98	0.89	0.88	1.15	0.89	1.05	1.20	1.27	1.01	0.99	1.38	1.20	0.95	1.00

Increased in low responders

Increased in high responders

B

Numerator

Denominator	3-Hydroxybutyric acid	Acetic acid	Acetone	Choline	Creatine	Creatinine	D-Glucose	Dimethyl sulfone	Formic acid	Glycine	Lactic acid	L-Alanine	L-Glutamine	L-Histidine	L-Isoleucine	L-Leucine	L-Lysine	L-Phenylalanine	L-Tyrosine	L-Valine
3-Hydroxybutyric acid	1.00	0.93	0.88	0.51	1.17	0.76	0.90	1.35	0.93	0.87	0.91	0.62	1.03	1.07	0.85	1.08	1.25	1.36	0.91	0.99
Acetic acid	1.08	1.00	0.92	0.86	1.29	0.83	0.69	1.02	0.81	0.89	1.12	0.84	0.96	1.39	1.04	0.92	1.52	0.80	1.01	1.01
Acetone	1.14	1.09	1.00	1.06	1.65	1.19	1.10	1.57	1.03	1.17	1.39	1.15	1.55	2.08	1.27	1.21	1.72	1.28	1.20	1.40
Choline	1.46	0.90	0.95	1.00	1.33	0.99	1.01	1.10	1.52	0.87	1.02	1.07	1.25	1.32	1.07	1.08	1.24	1.30	1.19	0.99
Creatine	0.85	0.78	0.61	0.64	1.00	0.80	0.70	0.97	0.90	0.88	0.71	0.87	0.96	1.10	0.79	0.84	1.07	0.84	0.89	0.77
Creatinine	1.31	1.21	0.84	0.98	1.25	1.00	1.06	1.13	0.89	0.94	0.93	1.02	1.13	1.47	1.09	1.15	1.68	1.23	0.99	0.95
D-Glucose	1.11	1.44	0.91	0.99	1.44	0.95	1.00	1.34	1.02	1.22	0.95	1.05	1.38	1.34	1.18	1.11	1.78	1.17	1.07	1.18
Dimethyl sulfone	0.74	0.98	0.64	0.88	1.03	0.88	0.75	1.00	1.10	0.90	0.82	0.99	1.23	1.41	0.89	0.89	1.39	0.88	0.92	0.89
Formic acid	1.08	1.24	0.97	0.65	1.11	1.13	0.98	0.91	1.00	0.99	0.98	1.15	1.26	1.17	0.86	1.11	1.85	1.42	1.22	0.89
Glycine	1.14	1.12	0.86	1.08	1.14	1.06	0.82	1.11	1.01	1.00	1.04	0.90	1.22	1.15	1.17	1.06	1.56	1.02	1.19	1.01
Lactic acid	1.09	0.90	0.72	1.09	1.41	1.08	1.05	1.22	1.02	0.96	1.00	1.13	1.34	1.56	1.25	1.14	1.87	1.28	1.10	1.11
L-Alanine	1.61	1.20	0.87	0.92	1.15	0.98	0.95	1.01	0.87	1.11	0.89	1.00	1.27	1.27	1.09	1.15	1.63	1.11	1.05	0.93
L-Glutamine	0.97	1.04	0.65	0.78	1.04	0.89	0.73	0.81	0.79	0.82	0.75	0.79	1.00	1.08	0.87	1.09	0.95	0.92	1.04	0.90
L-Histidine	0.93	0.72	0.48	0.56	0.91	0.68	0.75	0.71	0.85	0.87	0.64	0.79	0.93	1.00	0.84	0.73	0.80	0.73	0.83	0.76
L-Isoleucine	1.17	0.96	0.79	0.90	1.26	0.92	0.85	1.13	1.16	0.86	0.80	0.92	1.14	1.19	1.00	0.95	1.64	0.94	0.95	1.02
L-Leucine	0.93	1.09	0.79	0.90	1.19	0.87	0.90	1.12	0.90	0.94	0.88	0.87	0.92	1.36	1.05	1.00	1.67	0.89	0.97	0.86
L-Lysine	0.83	0.71	0.60	0.65	0.94	0.70	0.58	0.80	0.54	0.70	0.57	0.65	1.08	1.29	0.61	0.63	1.00	0.88	0.79	0.64
L-Phenylalanine	0.74	1.25	0.78	0.75	1.19	0.81	0.86	1.14	0.70	0.98	0.78	0.90	1.08	1.36	1.07	1.12	1.16	1.00	0.90	0.93
L-Tyrosine	1.10	0.99	0.83	0.76	1.12	1.01	0.93	1.09	0.82	0.84	0.91	0.95	0.96	1.20	1.05	1.03	1.26	1.12	1.00	0.96
L-Valine	1.01	0.99	0.71	0.83	1.31	1.05	0.85	1.12	1.12	0.99	0.90	1.07	1.11	1.32	0.98	1.17	1.63	1.08	1.04	1.00

Increased in low responders

Increased in high responders

Figure S3: Fold-change of high vs. low responders of metabolic ratios in A) Day 22, B) 3 Months. The changes in the metabolic ratios that were statistically significant using t-test (*p*-value<0.05) are in boxes. Color coding is based on conditional formatting of fold-change values.

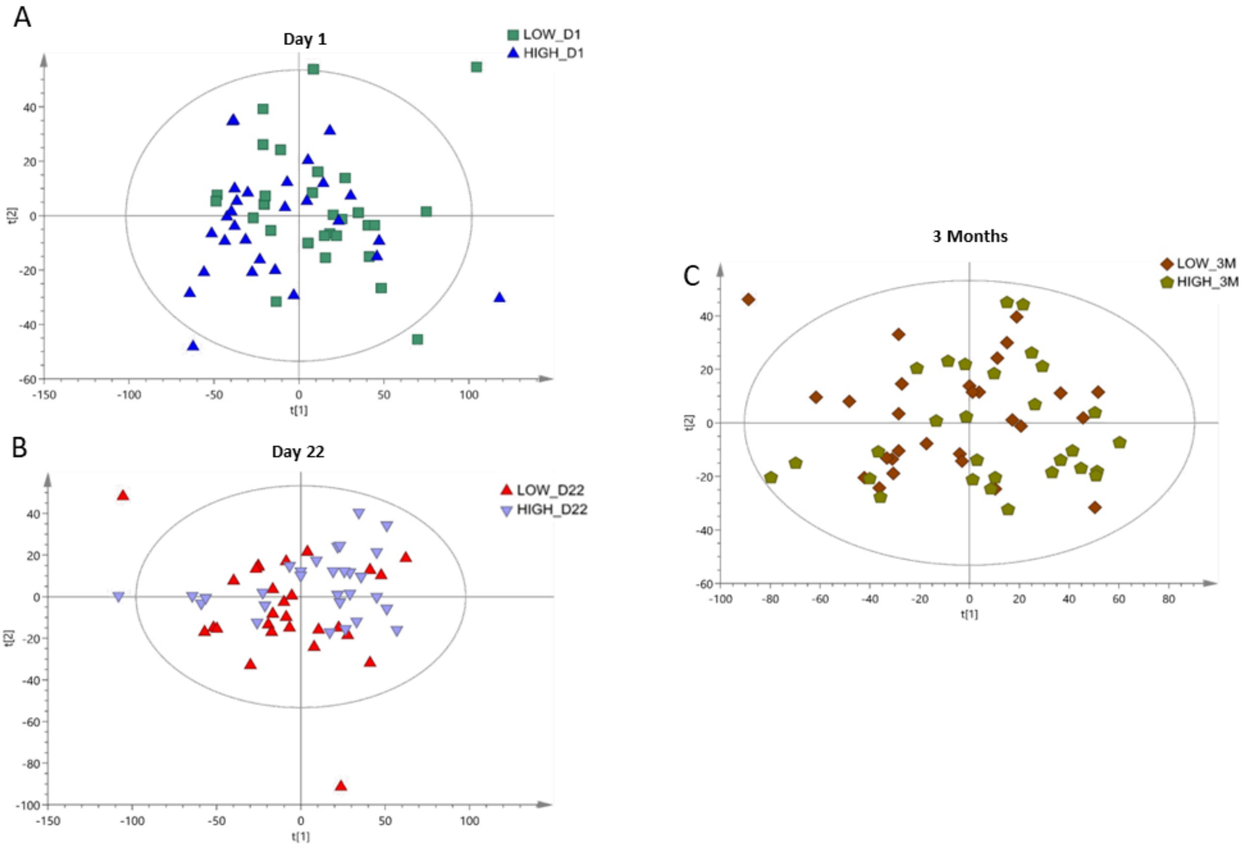

Figure S4: Scores plots obtained from the PCA of NMR CPMG spectra of plasma samples from high vs low responders at A) Day 1, B) Day 22, and C) 3 Months. It is observed that a trend for discrimination is already observed from Day 1, while this trend is diminished at 3 months. (■) Low responders at Day 1, (▲) High responders at Day 1, (▲) Low responders at Day 22, (▼) High responders at Day 22, (◆) Low responders at 3 months, (⬟) High responders at 3 months.

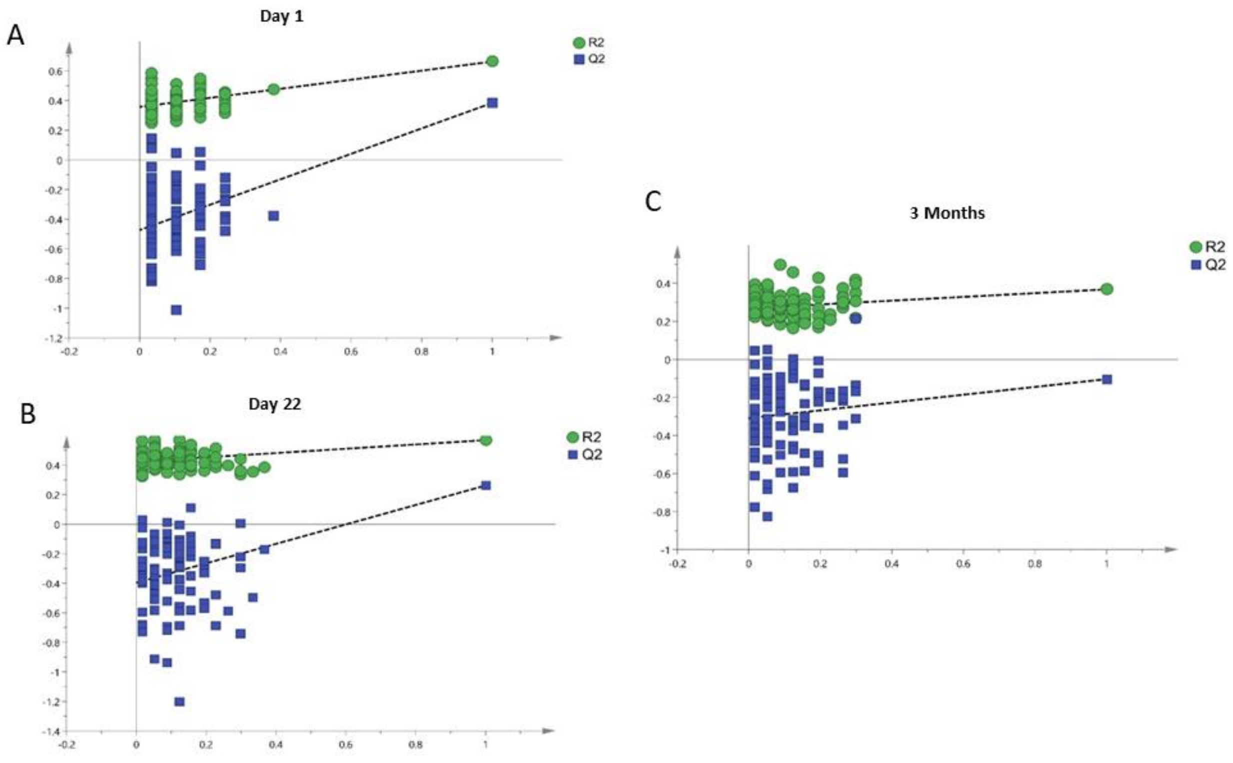

Figure S5: Permutation testing results from the OPLS-DA of NMR CPMG spectra from plasma samples of high vs low responders at A) Day 1, B) Day 22 and C) 3 Months. In order to assess the validity of the generated classifications, 100 random permutations were performed, and the quality parameters are plotted (on the left) along/together with R^2X and Q^2 of the original model (on the right). Validity of the Day 1 and Day 22 models was proved ("random" values lower than model parameters).

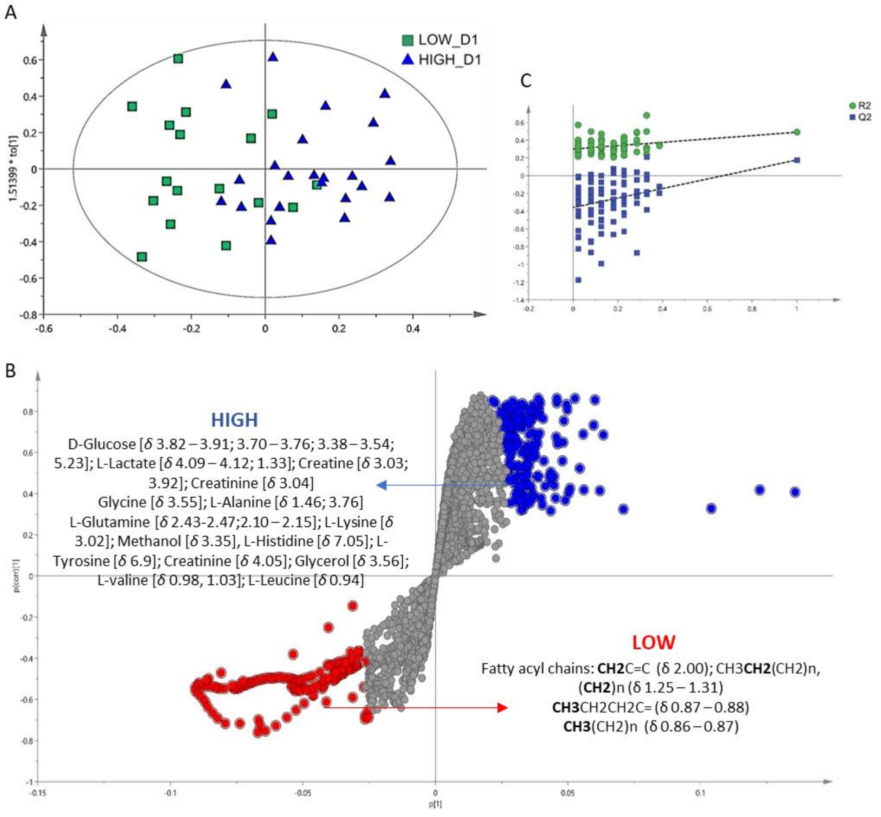

Figure S6: OPLS-DA of NMR CPMG from plasma samples of female high vs low responders at Day 1. A) Scores plot. The two groups are discriminated. (■) Low responders at Day 1, (▲) high responders at Day 1. B) S-plot. Spectral variables on the up- right corner (blue) are considered significantly increased in the high responders, while those on the down-left corner (red) are increased in the low responders. Metabolites with *p* and *p(corr)*~0 (coloured in grey) do not significantly impact the separation. Significant spectral variables and their assigned metabolites are shown. C) Permutation testing results (100 random permutations).

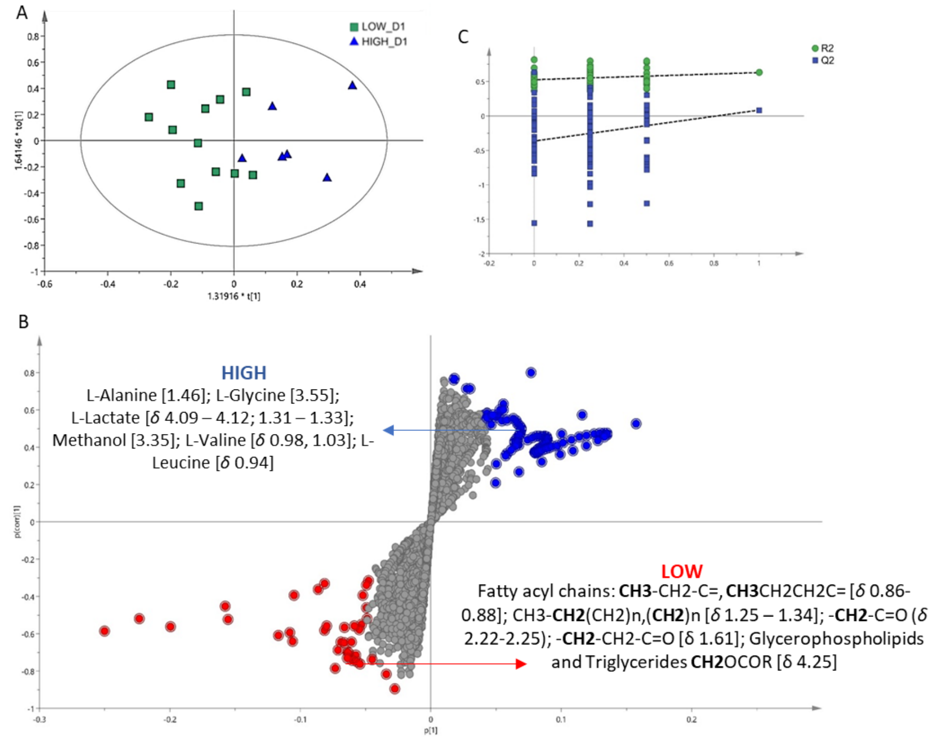

Figure S7: OPLS-DA of NMR CPMG from plasma samples of male high vs low responders at Day 1. A) Scores plot. The two groups are discriminated. (■) Low responders at Day 1, (▲) high responders at Day 1. B) S-plot. Spectral variables on the up- right corner (blue) are considered significantly increased in the high responders, while those on the down-left corner (red) are increased in the low responders. Metabolites with *p* and *p(corr)*~0 (coloured in grey) do not significantly impact the separation. Significant spectral variables and their assigned metabolites are shown. C) Permutation testing results (100 random permutations).

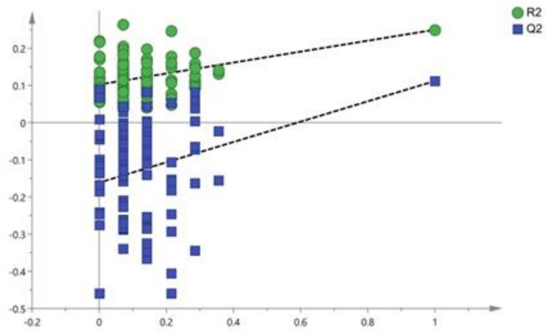

Figure S8: Permutation testing results (100 random permutations) from the OPLS-DA of NMR LED spectra.

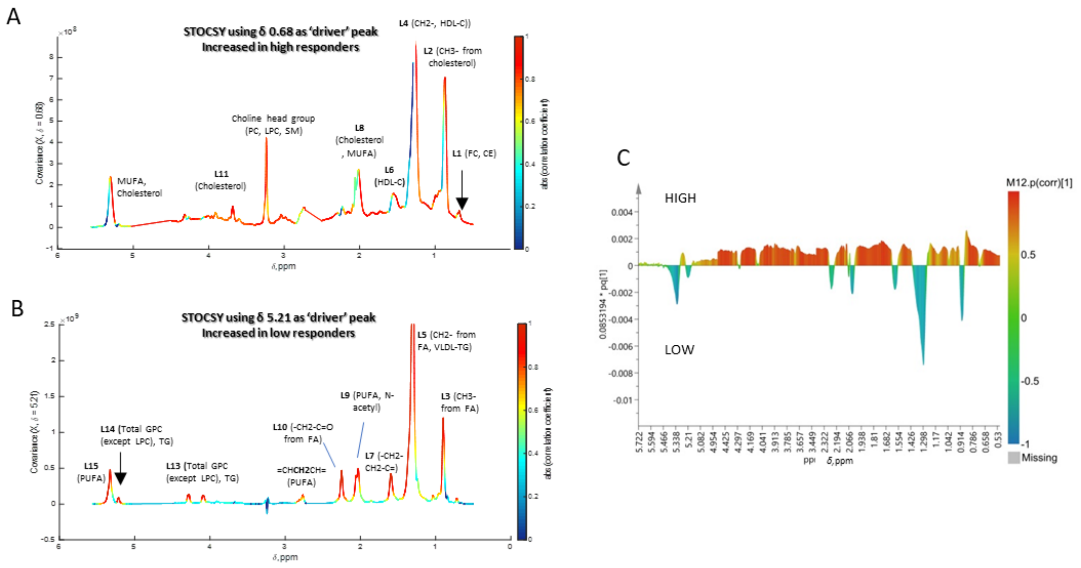

Figure S9: STOCSY plot using A) δ=0.68 (increased in the high responders) and B) δ=5.21 (increased in the low responders) as "driver peaks" [shown with an arrow]. Signal assignments were based on the fact that spectral features exhibiting high correlation belong on the same molecule or lipid class. Numbering of lipids is the same as in Figure 4A. C) Loadings plot (in line format) from the OPLS-DA of NMR LED spectra from plasma samples of high vs low responders after first dose. In red spectral variables increased in the high responders and in blue increased in the low responders group. Coloring based on the correlation p(corr). The pq1 value refers to the weight that combines the X and Y loadings (p and q).

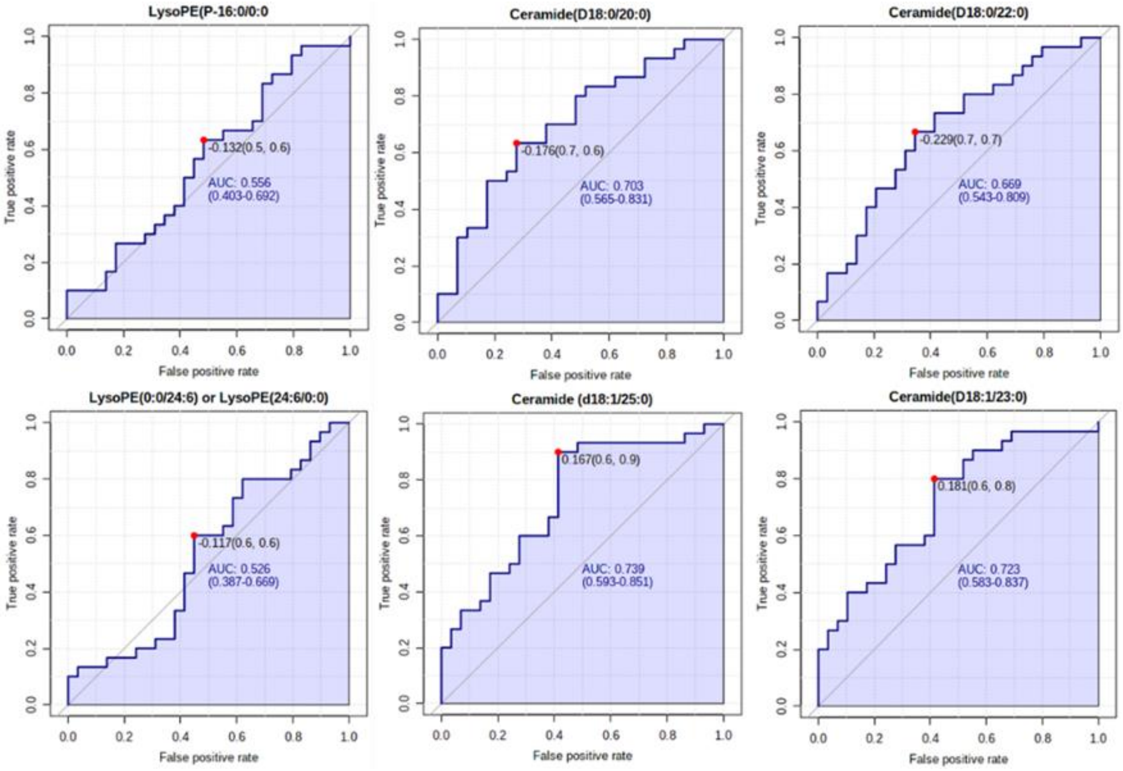

Figure S10: ROC curves of the annotated features.

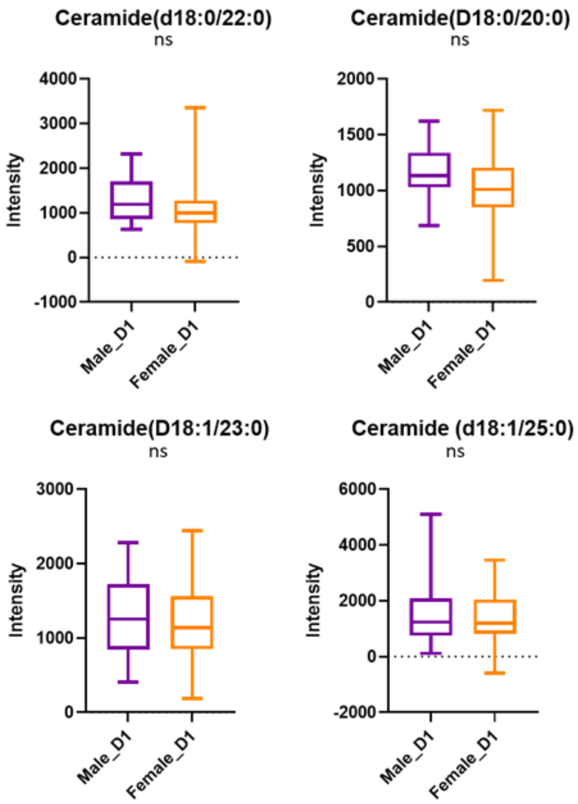

Figure S11: Box and whiskers plots of the detected ceramides for male vs female at Day 1. The differences were not statistically significant; *p*-values >0.05. ns: not significant.

Supplementary S2.

3 Materials and Methods

3.1 Chemicals and Reagents

The chemicals that were used for NMR and UPLC-MS analysis are descripted in Supplementary A. Deuterium oxide was purchased from Deutero GmbH (Kastellaun, Germany). Trimethylsilyl propionate (TSP), sodium phosphate dibasic (Na_2HPO_4) and hydrochloric acid (HCl) 37% were purchased from Sigma-Aldrich-Merck (Darmstadt, Germany). Sodium azide (NaN_3) was purchased from Acros Organics-Thermo Fisher Scientific (Waltham, Massachusetts, U.S.). All chemicals were of analytical grade and dissolved in ultrapure water.

For the MS analysis, methanol was obtained from Fisher Scientific (Loughborough, UK) and formic acid was purchased from Carlo Erba reagents (Val de Reuil Cedex, France). Ultra-pure water was produced by a Millipore Direct-Q System (Molsheim, France). All solvents were of LC–MS grade of purity. The three internal standards (ISTD) reserpine, yohimbine and 4-aminosalicylic acid were purchased from Sigma-Aldrich (Steinheim, Germany).

3.2 Sample preparation

3.2.1 NMR

Plasma samples were stored at −80 °C. At the day of analysis samples were left to thaw on ice. In case of particulates in the sample, they were removed using a needle. 350 μL of plasma were transferred to an Eppendorf tube and diluted to 700 μL with phosphate buffer pH=7.4, containing 0.074 M Na_2HPO_4, 0.8 mg mL^{-1} TSP as internal standard and 0.4 mg mL^{-1} NaN_3 as conservative. The pH of the buffer was adjusted using 1N HCl. No vortexing was applied, and mixing was achieved with gentle pipetting. An aliquot of 600 μL was drawn and transferred to a 5-mm-diameter NMR tube. A blank solution containing 350 μL of the phosphate buffer solution and 350 μL of ultrapure water was also prepared.

3.2.2 MS

For the UPLC-MS analysis, the plasma samples were prepared according to the following sample preparation protocol. For each sample, a 200 μL plasma aliquot was placed into a labelled 1.5 mL Eppendorf tube and 600 μL of methanol was added for protein precipitation. Then, the samples were vortexed for 15 s and centrifuged at 15,000g for 15 min at 4 °C. An aliquot of 185 μL of the supernatant was transferred into labelled 1.5 mL Eppendorf tube and finally evaporated in the Hypervac concentrator for 120 min at room temperature. Consequently, 100 μL of water containing the three ISTDs (1 μg mL^{-1} of each ISTD) was added to each dry sample, vortexed for 15 s, centrifuged at 15,000g for 15 min and transferred to 200 μL inserts placed in appropriate screw-capped autosampler vials.

3.3 Data Acquisition

3.3.1 NMR Data Acquisition

For each sample one standard ^1H 1D experiment with water suppression using the NOESY1D-pre-saturation pulse sequence with magnetic field gradients, as well as a ^1H 1D T2-edited spectrum using the Carr-Purcell-Meiboom-Gill (CPMG) sequence and its complementary ^1H 1D T1-edited spectrum using the longitudinal encode-decode (LED) sequence were obtained. The NOESY1D-pre-saturation pulse sequence results in a spectrum with both the sharp peaks of small compounds and the broad bands of macromolecules (e.g., lipoproteins and proteins), while the CPMG experiment diminishes the intensities of the broad signals using the variation in the nuclear spin relaxation times between macromolecules and small compounds. That results in a spectrum of slow relaxing signals with improved baseline, where the sharp peaks of the metabolites are easier to be quantified. On the other hand, the LED pulse sequence reduces the intensities of the small molecules resulting in a spectrum mainly consisting of the broad protein signals and thus lipoprotein alterations can be detected. For each NOESY and LED spectrum, 32 scans were acquired with a spectral width of SW = 18,028.846 Hz and a sampling of 96,000 data points, resulting in an acquisition time of 2.7 sec. For the CPMG spectrum, 32 scans were acquired with a spectral width of SW = 12,019.230 Hz and a sampling of 73,000 data points, resulting in an acquisition time of 3.1 sec. A mixing time of 10 msec was used in the NOESY pulse sequence. All the experiments were carried out at a temperature of 310 K. Sample loading, temperature stability, field homogeneity, pulse calibration, data acquisition, and processing (including phase, baseline correction and axis calibration) were fully automated and controlled by the IconNMR v. 5.0.7 software (Bruker BioSpin GmbH, Rheinstetten, Germany). TopSpin 4.0.9 (Bruker BioSpin GmbH, Rheinstetten, Germany) was used for spectra visualization and chemical shift calibration to the anomeric ^1H proton of α-glucose at 5.23 ppm from TSP. For a selected sample, Total Correlation Spectroscopy (TOCSY) and Edited Heteronuclear Single Quantum Coherence (HSQC) 2D NMR experiments were performed to aid molecular identification.

3.3.2 LC – MS Data Acquisition

3.3.2.1 Instrumentation

For the MS analysis, a Waters ESI-QTOF Premier mass spectrometer (Waters Corp., Milford, CT, USA) coupled to an Acquity UPLC^{TM} system (Waters Corp., Milford, MA, USA) was used. The Ultra High Performance Liquid Chromatography (UPLC) system was equipped with a binary solvent manager system, an electronically controlled oven and a sample manager. The chromatographic separation was performed on a Waters Acquity BEH C_{18} (2.1 × 100 mm, 1.7 µm, Waters Corp., Milford, CT, USA) analytical column. Centrifugation of the plasma samples during the sample pretreatment stage was performed with anIEC Micromax RF centrifuge (Thermo Fisher Scientific, Waltham, MA, USA) and their

evaporation was performed with a HyperVac™ centrifugal vacuum concentrator (GYROZEN, Gochon-eup, Gimpo-si,Gyeonggi-do, Rep. of KOREA).

3.3.2.2 UPLC conditions

The mobile phase consisting of 0.01% formic acid/water (*v/v*) (solvent A) and methanol (solvent B) for positive ion mode and 5mM aqueous ammonium formate (solvent A) and methanol (solvent B) for the negative ion mode. The elution program was as follows: 1 min at 2% B; from 2% B to 100% B in 15 min; 4 min at 100% B; from 100% B to 2% in 2 min; equilibration at 2% B for 2 min. The flow rate was 0.4 mL min^{-1} and the total analysis time was 24 min. The column temperature was maintained at 50 °C, while the sample manager temperature was kept at 8 °C. The injection volume was 5 µL.

3.3.2.3 HRMS conditions

For the positive ion mode (ESI+), the capillary voltage, the sample cone voltage, the extraction cone voltage and the ion guide were kept at 2.5 kV, 35 V, 4.0 V and 2.0 V, respectively. For the negative ion mode (ESI-), the capillary voltage, the sample cone voltage, the extraction cone voltage and the ion guide were kept at 1.5 kV, 35 V, 3.0 V and 5.0 V, respectively. In both ion modes, the desolvation gas (nitrogen) flow and temperature were 650 L hr^{-1} and 350 °C, respectively. The source temperature was 120 °C and the MCP plates were operated at 1950 V. The TOF analyser was operated in the V optics mode, affording a resolution of 9,000. A full scan mode over the range of 50–1000 Da was selected and the spectra were acquired in centroid mode. The collision gas was argon. For the acquisition of the full-scan mass spectra the collision energy was set at 2 V, while for the tandem MS analysis, a collision energy ramp from 20 to 40 V was used. The scan time was 0.2 s for the full-scan spectra and 0.4 s for the MS/MS, while the inter scan delay was 0.02 s for both functions. In order to improve the mass accuracy, a lock mass compound (1 µg mL^{-1} of reserpine; *m/z* 609.2812 in positive mode and *m/z* 607.2656 in negative mode) was continuously infused at an 8 µL min^{-1} flow rate using the built-in syringe pump of the instrument. The MassLynx V 4.1 (SCN 907) software (Waters Corp., Milford, MA, USA) was used for instrumentation control and data acquisition.

3.3.2.4 Quality control samples

A pooled sample to serve as quality control (QC) was prepared by mixing equal aliquots (20 µL) from the supernatants of every studied sample. To check for the instrumental stability and performance, four QC samples were injected at the beginning of the analytical batch, one QC every five samples throughout the run and four QC samples at the end of the batch. Software-based correction has been applied to the data, employing data normalization algorithms.

3.4 Data Processing

3.4.1 NMR Processing

3.4.1.1 NMR Data Pre-processing

Spectral data were imported into MATLAB [Version 8.3 (R2014a Mathworks Inc., Natwick, MA, USA] for further processing. Reduction of the number of variables from the full resolution CPMG and LED NMR spectra (>60000 features) was performed by bucketing of the NMR spectra. A spectral bucketing of 0.001 was applied at the range of δ = 0.5– 8.5 ppm resulting in 8001 spectral bins. Due to the fact that blood collection was performed in prefilled vials with either EDTA or citric acid as anticoagulant agents, the strong resonance lines of EDTA and citric acid hindered the analysis and have been excluded from the NMR dataset. Overall, the following regions were removed: 1) δ4.35–5.00 corresponding to water region, 2) δ 2.47–2.93 corresponding to citric acid, Ca-EDTA (δ 2.53–2.59) and Mg-EDTA (δ 2.67–2.71) peaks 3) δ 3.06–3.03 corresponding to Ca-EDTA, Mg-EDTA and EDTA peaks and 4) δ 3.57–3.68 corresponding to EDTA peaks resulting in 6555 spectral bins. The remained spectral regions were normalized to total intensity using the remaining spectral data points. For LED spectra, a spectral bucketing of 0.001 was applied at the range of δ = 0.5–6.0 ppm resulting in 4021 spectral bins after removal of water and anticoagulant regions.

3.4.1.2 NMR Metabolite Identification

For metabolites that pose more than one of the following spectral characteristics: relatively high intensities /concentration, peaks in various chemical shifts /existence-presence of non-equivalent protons, distinct peak shapes /patterns and no overlapping with other signals, leading to a specific "fingerprint" of the metabolite, the identification was based on simple inspection of the ^{1}H NMR spectrum and database search (e.g., glucose, lactic acid etc.). The Chenomx NMR Suite (Chenomx, Edmonton, Canada), a software that includes standard metabolite spectra, aided molecular identification substituting the manual matching. Whenever the inspection of the ^{1}H 1D NMR spectra did not provide an adequate confidence level of annotation, 2D NMR experiments on the selected samples were exploited. With 2D NMR experiments, information is provided about the coupling pattern JRES), the spin systems–(TOCSY), as well as the ^{1}H - ^{13}C correlation (HSQC). Finally, information on related features that arise from the same metabolite were identified through the use of statistical spectroscopy techniques (Statistical Total Correlation Spectroscopy, STOCSY) performed in MATLAB. STOCSY is based on the fact that signals belonging to the same molecules are characterized by linear relationships of the NMR peak intensities across the spectra. The algorithm analyses the covariance of a given spectral variable with all the NMR features and calculates the correlation matrix depicted as a pseudo- two- dimensional NMR spectrum that displays the degree of correlation (e.g., the correlation coefficient) of each peak with the variable of interest ("driver peak"). This pseudo-

spectrum is then compared with publicly available databases and prior in-house knowledge, as well as with published data on human plasma metabolomics.

3.4.1.3 NMR Targeted Metabolite Quantification

A computationally derived experiment that reduces macromolecular signals directly from the NOESY ^{1}H 1D-NMR spectrum called "SMolESY" (Small Molecule Enhancement SpectroscopY) was also performed. SMolESY increases the resolution of small molecules and depletes macromolecular signals by calculating the first partial derivative of the imaginary data of the NOESY spectrum, and since it does not rely on T2 constant modulation unlike the CPMG and LED experiments, the inherent quantitative quality of the conventional 1H-NMR spectrum is preserved. "SMolESY-select" an algorithm for metabolite panel measurement of 22 important metabolites in plasma/serum profiles was used for quantification. [1]

3.4.1.4 NMR Statistical Analysis

The association between the metabolite levels at Day 1 to the vaccine-induced immune response of individuals was examined by measuring the correlation of the untargeted NMR spectral bins (metabolic features) with the NAbs levels of individuals (response). High correlation means that the metabolic trait and the response have a strong relationship with each other, while a low correlation means that these are possibly not related. Since only individuals with NAbs levels at the two extremes of their distribution were included in the analysis, response was divided into "Low" and "High" (categorical outcome). Spearman's correlation was performed, as its coefficient measures the monotonic relationship between two continuous or ordinal variables and is suitable for ordered categorical data, nonlinear relationships, non-normal distributions, and extreme values. Partial correlation was performed to control for the effect of confounding variables, using the 'partialcorr' function in MATLAB. Analysis was adjusted for age and gender. Associations were tested for 6555 normalized, log-transformed (log10) NMR bins after the removal of the water and anticoagulant regions. Since thousands of spectral bins were simultaneously tested, two main approaches for multiple comparisons correction (i.e., minimize the chance to get a false positive result) were applied: a) the stricter Bonferroni family-wise error rate and b) the false discovery rate (FDR) using the Storey procedure. For 95% confidence, the Bonferroni threshold was defined as 0.05/n, where n is the number of metabolites tested for association and the FDR threshold was estimated in MATLAB. The 6555 NMR spectral bins versus the -log10(*p*-value) were plotted using MATLAB, creating a pseudo-spectrum that visualizes the degree of association. For each associated metabolite we selected the 'sentinel' feature with the lowest *p*-value at Day 1 and further tested for statistical significance in other pairwise comparisons (e.g., Low responders at Day 1 versus Low responders at 3 months) using

Analysis of Variance (ANOVA). ANOVA and the corresponding boxplots were created using Graphpad Prism 8 (GraphPad Software, Inc.).
Finally, all possible ratios of metabolite concentrations derived from SMolESY (20 x 20=400 traits) were calculated in R. Fold-change of the median and *p*-value from t-testing was further calculated for each metabolic ratio between the two examined groups on Day 1.

3.4.2 LC – MS Processing

3.4.2.1 Software

Data preprocessing was performed employing Proteowizard (https://proteowizard.sourceforge.io/, accessed on 12 February 2022), MZmine 2.53 (http://mzmine.github.io/, accessed on 12 February 2022), RStudio (https://www.rstudio.com/products/rstudio/, accessed on 12 February 2022), the web-based MetaboAnalyst5.0 suite (https://www.metaboanalyst.ca/, accessed on 12 February 2022), GraphPad Prism 8 and Microsoft Excel 2016. Furthermore, MS-Dial 4.70 (http://prime.psc.riken.jp/compms/msdial/main.html, accessed on 12 February 2022) and MS-Finder 3.51 (http://prime.psc.riken.jp/compms/msfinder/main.html, accessed on 12 February 2022) programs were also employed.

3.4.2.2 LC – MS Data Pre-processing

For the LC-MS analysis, a data-independent acquisition (DIA) method was followed for strengthening the structural determination by MS/MS. In the broadband DIA method, all ions that enter the mass spectrometer at a given time are fragmented and analyzed in the second stage of tandem mass spectrometry. Since all precursor ions in a survey scan are fragmented, there is the need to incorporate post-acquisition *in silico* data processing steps to deconvolute the resulting complex fragment ion spectra. Through the spectrum deconvolution, MS/MS information is revealed for each precursor ion, enabling the subsequent feature identification by comparing to the experimental MS/MS spectra deposited in the corresponding databases. MS-Dial is a universal program for untargeted metabolomics that apart from the peak picking preprocessing steps, performs spectra deconvolution for DIA data. The peak picking preprocessing steps include peak detection, chromatogram deconvolution, deisotoping and peak list alignment. These procedures can also be performed in MZmine 2.53. The advantage of the MZmine is that the user can visually check the selected parameters for each procedure, while the MS-Dial program does not have a preview choice for each step. The drawback is that the spectra deconvolution mode is not included in MZmine. Thus, a combination of the two pieces of software was used for the MS data processing.

For the evaluation of the MZmine results, the presence of the ISTDs in all the analyzed samples including the QC samples was deemed as the criterion of the successful peak picking procedure. The *m/z* values and retention times for reserpine, yohimbine and 4-aminosalicylic acid were found in ESI

(+): *m/z* 154.0500 at 2.84 min, *m/z* 355.1970 at 6.33 min and *m/z* 609.2760 at 9.76 min, respectively, and in ESI (-) *m/z* 152.0360 at 0.76 min, *m/z* 353.1873 at 8.00 min and *m/z* 607.2662 at 12.20 min, respectively. Selecting those ISTDs, the *m/z* and retention time ranges were covered for all the span of the mass range and the chromatographic time.

Proteowizard was used to convert the instrument native RAW files to the MZML data format and following this, the data were imported to MZmine 2.53 and processed applying the peak detection, deconvolution, deisotoping and alignment procedures. All the parameters used for these processes in MZmine are summarized in **Table S6.** The generated peak list (accurate mass – t_R vs. area) was exported as CSV file to Microsoft Excel 2016 and manipulated appropriately using the CONCATENATE, ROUND and TRANSPOSE commands. The missing values imputation was performed using Metaboanalyst 5.0.

Table S6: MZmine parameters for the data preprocessing of plasma samples from both (+) and (-) ESI-UPLC-HRMS analyses.

**MZ mine procedure**		**ESI (+)**	**ESI (-)**
Crop filter	Retention time (min)	0 – 21.5	0 – 21.5
Mass Detection	Algorithm	centroid	centroid
	Noise Level	9.0E0	4.5E0
ADAP Chromatogram builder	Min group size in # of scans	3	3
	Group intensity threshold	1.0E2	5.0E1
	Min highest intensity	2.0E1	5.0E1
	m/z tolerance (ppm)	5	5
Chromatogram deconvolution	Algorithm	Baseline cut-off	Baseline cut-off
	Min peak height	5.0E2	5.0E1
	Peak duration range (min)	0.01 – 0.75	0.01 – 0.75
	Baseline level	5.0E0	5.0E0
Deisotoping	Algorithm	Isotopic peaks grouper	Isotopic peaks grouper
	m/z tolerance (ppm)	5	5
	Retention time tolerance (min)	0.15	0.15
Identification of adducts	Algorithm	Join aligner	Join aligner
Alignment	*m/z* tolerance (Da)	5	5
	Weight for *m/z*	1	1
	Retention time tolerance (min)	0.2	0.2
	Weight for RT	1	1
Duplicate peak filter	Filter mode	New average	New average
	m/z tolerance (Da)	5	5
	Retention time tolerance (min)	0.02	0.02

Prior to the multivariate statistical analysis, three normalization methods implemented in R were evaluated. The CCMN and the NOMIS algorithms, implemented to “normalise” R package (https://cran.r-project.org/web/packages/metabolomics/index.html, accessed on 12 February 2022) are based on multiple internal standard based normalization and the QC-RLSC algorithm implemented to the R package “statTarget” (https://bioconductor.org/packages/release/bioc/html/statTarget.html, accessed on 12 February 2022) uses the QC-based locally weighted scatter-plot smoothing method to alleviate the effects of signal drift.

MS-Dial was employed for peak detection, chromatogram deconvolution, deisotoping, peak list alignment and spectra deconvolution of data-independent MS/MS. Similar parameters with those in MZmine analysis were used. ReifycsAbf (Analysis Base File) converter (https://www.reifycs.com/AbfConverter/, accessed on 12 February 2022) was used to convert the instrument native RAW files to the ABF data format. All the parameters are summarized in **Table S7.**

Table S7: MS-Dial parameters for the data preprocessing of plasma samples from both (+) and (-) ESI-UPLC-HRMS analyses.

**MS Dial procedures**		**ESI (+)**	**ESI (-)**
Mass accuracy	MS1 tolerance (Da)	0.01	0.01
	MS1 tolerance (Da)	0.1	0.1
Peak detection parameters	Min peak height	90	100
	Mass slice width (Da)	0.1	0.1
	Smoothing method	Linear weighted moving average	Linear weighted moving average
	Smoothing level (scans)	3	3
	Minimum peak width (scans)	5	5
MS2 deconvolution parameters	Sigma window value	0.5	0.5
	MS/MS abundance cut-off	5	0
	Keep the isotopic ions until (Da)	0.33	0.33
Identification	MSP file	MSMS-Public-Pos-VS15*	MSMS-Public-Neg-VS15*
	Retention time tolerance (min)	200	200
	Accurate mass tolerance MS1 (Da)	0.01	0.01
	Accurate mass tolerance MS2 (Da)	0.1	0.1
	Identification score cut-off (%)	80	80
Adducts			
Alignment	Retention time tolerance (min)	0.05	0.05
	MS1 tolerance (Da)	0.01	0.01

*Available on http://prime.psc.riken.jp/compms/msdial/main.html#MSP (accessed on 5 January 2022).

MS Metabolite Identification

Metabolite identification was performed using free-available MS spectra databases including (a) the Human Metabolome Data Base (HMDB) (https://hmdb.ca/, accessed on 12 February 2022), (b) the METLIN Metabolomics Database (https://metlin.scripps.edu, accessed on 12 February 2022), (c) the MassBank database (http://www.massbank.jp/, accessed on 12 February 2022) and (f) the LIPID MAPS (http://www.lipidmaps.org, accessed on 12 February 2022), allowing a mass error < 20 ppm. Ion adducts were also taken into consideration. MS-Finder was used for the feature identification, selecting the spectral database search and the formula prediction and structure elucidation by *in silico* fragmentation options. The internal experimental libraries MassBank, GNPS and ReSpect were selected. All the parameters are summarized in **Table S8**.

Table S8: MS-Finder parameters for the feature identification.

MS-Finder Procedures		Values
Mass tolerance setting	Mass tolerance MS1 (Da)	0.01
	Mass tolerance MS2 (Da)	0.1
Abundance setting	Relative abundance cut-off (%)	10
Formula calculation setting	Isotopic ratio tolerance (%)	40
	Element ratio check	Common range
	Element selection	O, N, P, S
	Maximum report number	10
	Time out (min)	1
In silico MS/MS	Tree depth	2

3.4.3 NMR and LC – MS Multivariate Statistical Analysis

PCA and Orthogonal Partial Least Square-Discriminant Analysis (OPLS-DA) were applied in the binned, normalized to total intensity (excluding EDTA and Citric acid spectral regions) NMR data and in LC – MS data, using SIMCA-P 14.0 (Umetrics, Umea, Sweden). For the supervised methods (PLS-DA and OPLS-DA) the quality of obtained models was assessed via R^2X (variance explained by X Matrix) and Q^2 (goodness of prediction) obtained by 7x cross-validation. Permutation testing was performed using 100 random permutations and the corresponding quality parameters were calculated and compared with those of the original model to assess the quality of the generated classification. Data were either pareto-scaled or scaled to unit variance, whichever led to higher values of quality parameters. Selection of significant variables in the OPLS-DA analysis was based on the S-plot, where the covariance (x-axis) and correlation (y-axis) indicate the contribution and the confidence of contribution, respectively, for each spectral variable in the observed discrimination. For the MS analysis, only variables with VIP values (Variable importance in projection) > 1.5 were taken into consideration.
